# Supplementary material for: Ribosome heterogeneity in Drosophila melanogaster gonads through paralog-switching
Source: Nucleic Acids Res. 2021 Jul 20;50(4):2240–57. doi: 10.1093/nar/gkab606 (PMC8887423; doi:10.1093/nar/gkab606)
Supplement: gkab606_Supplemental_Files [file gkab606_supplemental_files.zip › Hopes_etal_Sup1.pdf]

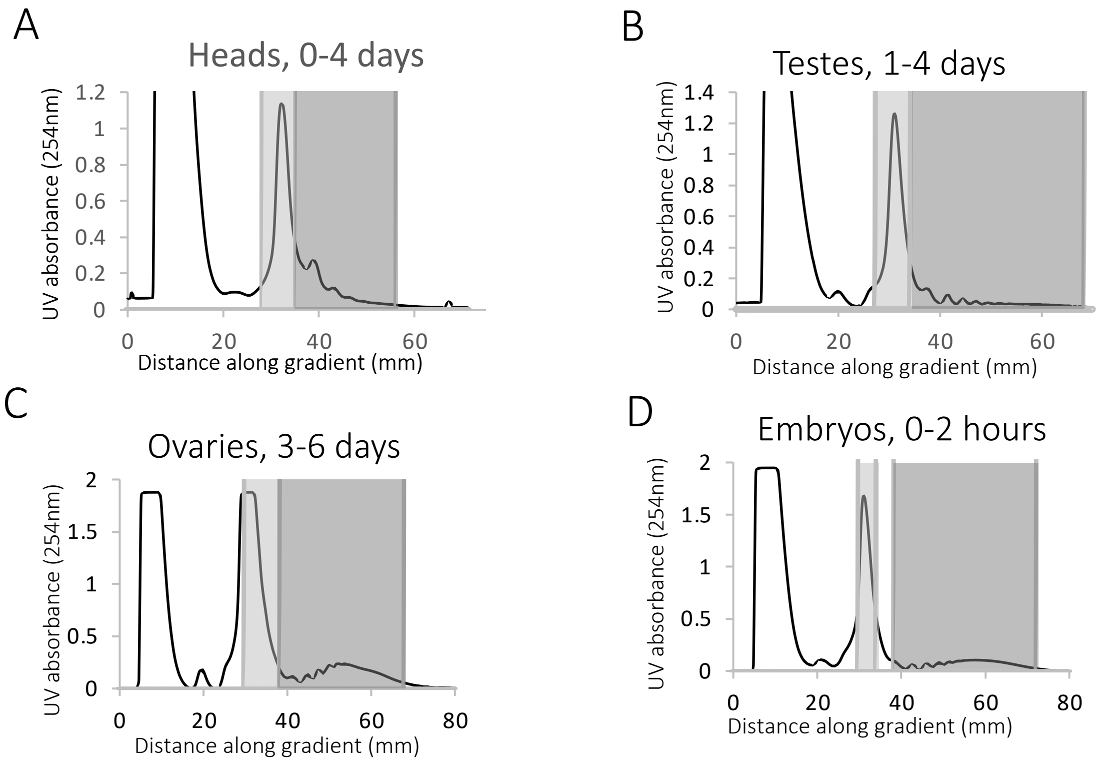

Sup 1: Determined ribosomal composition in tissues and during development

254 nm UV plots across sucrose gradients with 80S and polysomal complexes isolated from (A) 50:50 mixture of female:male 0-3 day old heads. (B) ~500 pairs of 1-4 day old adult testes, (C) ~500 pairs of 3-6 day old adult ovaries, (D) 0-2 hour embryos. Light grey shading indicates fractions used for 80S and dark grey for polysomes.
