# Supplementary material for: Ribosome heterogeneity in Drosophila melanogaster gonads through paralog-switching
Source: Nucleic Acids Res. 2021 Jul 20;50(4):2240–57. doi: 10.1093/nar/gkab606 (PMC8887423; doi:10.1093/nar/gkab606)
Supplement: gkab606_Supplemental_Files [file gkab606_supplemental_files.zip › Hopes_etal_Sup2.pptx]

## Slide 1
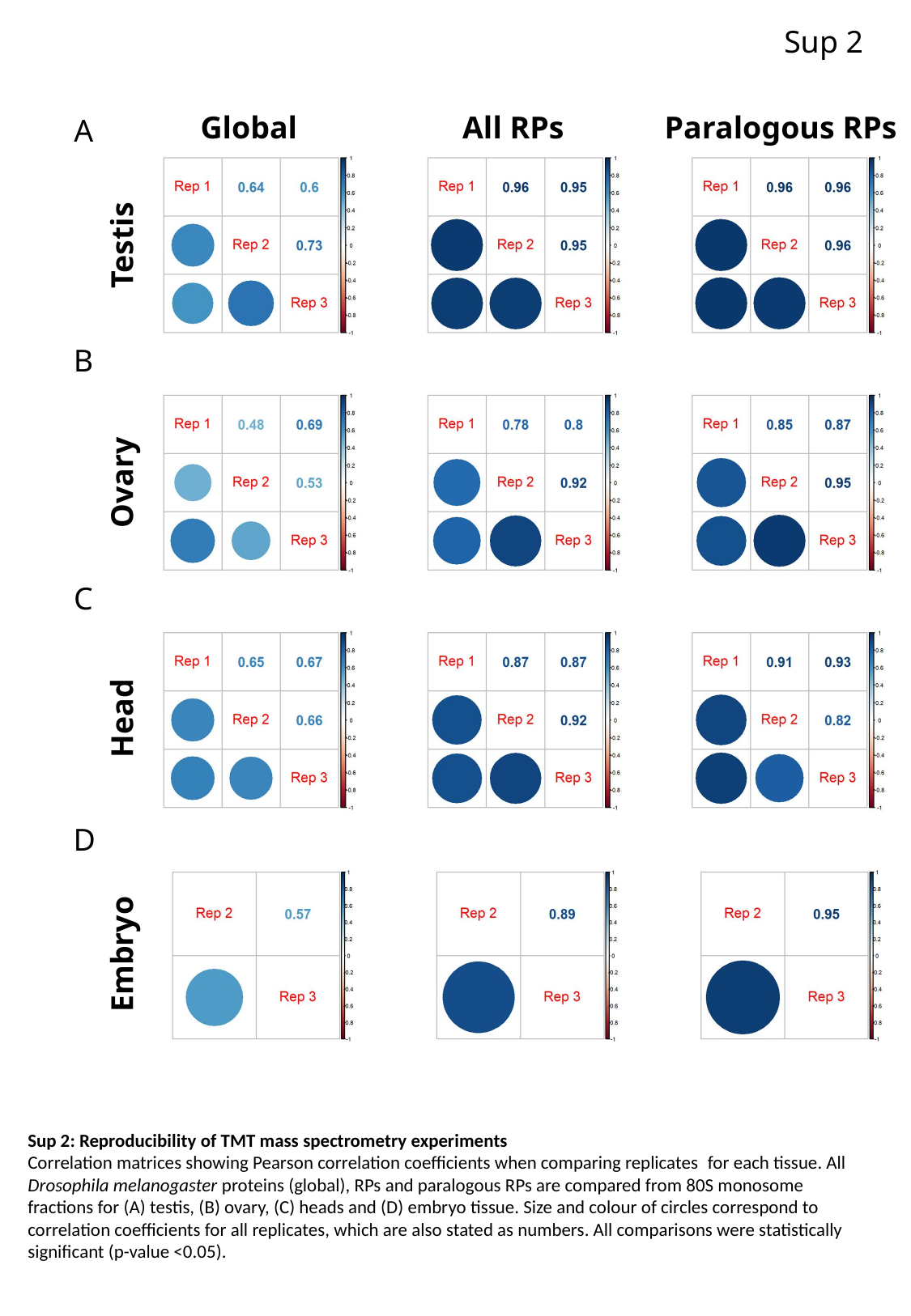

Sup 2
All RPs
Paralogous RPs
Global
A
Testis
B
Ovary
C
Head
D
Embryo
Sup 2: Reproducibility of TMT mass spectrometry experiments
Correlation matrices showing Pearson correlation coefficients when comparing replicates  for each tissue. All Drosophila melanogaster proteins (global), RPs and paralogous RPs are compared from 80S monosome fractions for (A) testis, (B) ovary, (C) heads and (D) embryo tissue. Size and colour of circles correspond to correlation coefficients for all replicates, which are also stated as numbers. All comparisons were statistically significant (p-value <0.05).
