# Supplementary material for: Ribosome heterogeneity in Drosophila melanogaster gonads through paralog-switching
Source: Nucleic Acids Res. 2021 Jul 20;50(4):2240–57. doi: 10.1093/nar/gkab606 (PMC8887423; doi:10.1093/nar/gkab606)
Supplement: gkab606_Supplemental_Files [file gkab606_supplemental_files.zip › Hopes_etal_Sup3_4.pptx]

## Slide 1
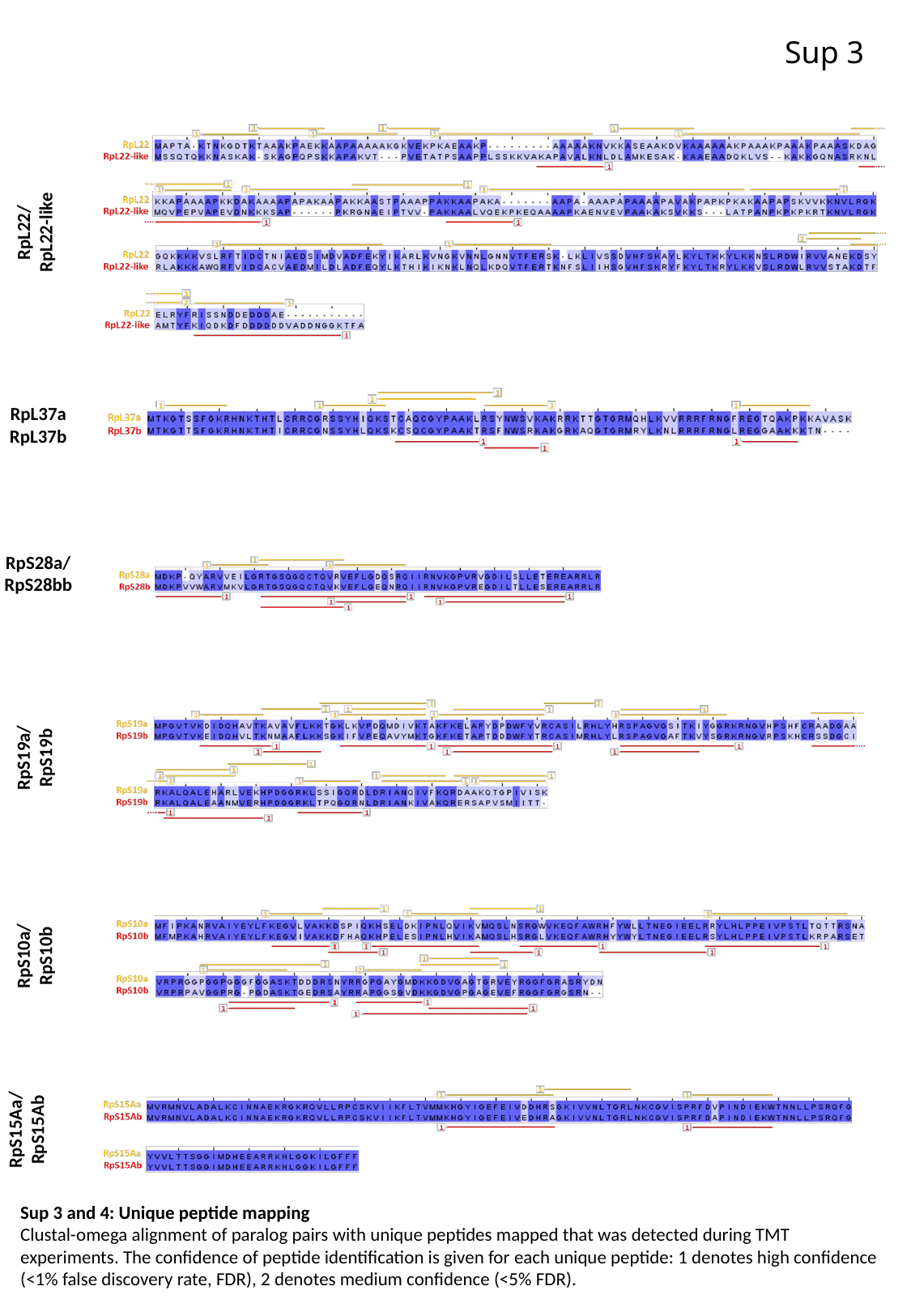

Sup 3
RpL22/
RpL22-like
RpL37a
RpL37b
RpS28a/
RpS28bb
RpS19a/
RpS19b
RpS10a/
RpS10b
RpS15Aa/
RpS15Ab
Sup 3 and 4: Unique peptide mapping
Clustal-omega alignment of paralog pairs with unique peptides mapped that was detected during TMT experiments. The confidence of peptide identification is given for each unique peptide: 1 denotes high confidence (<1% false discovery rate, FDR), 2 denotes medium confidence (<5% FDR).

## Slide 2
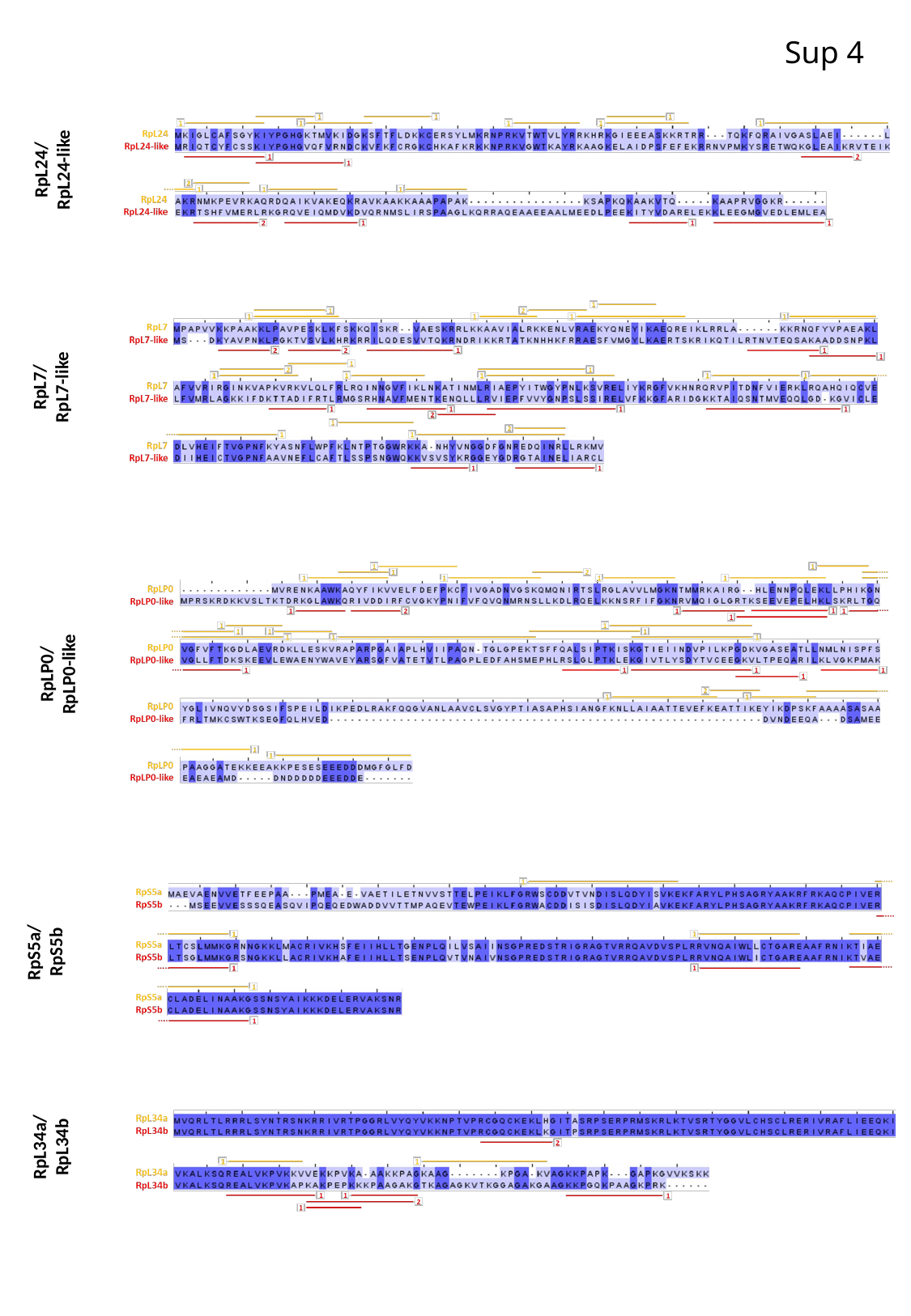

Sup 4
RpL24/
RpL24-like
RpL7/
RpL7-like
RpLP0/
RpLP0-like
RpS5a/
RpS5b
RpL34a/
RpL34b
