# Supplementary material for: Ribosome heterogeneity in Drosophila melanogaster gonads through paralog-switching
Source: Nucleic Acids Res. 2021 Jul 20;50(4):2240–57. doi: 10.1093/nar/gkab606 (PMC8887423; doi:10.1093/nar/gkab606)
Supplement: gkab606_Supplemental_Files [file gkab606_supplemental_files.zip › Hopes_etal_Sup_7_8.pptx]

## Slide 1
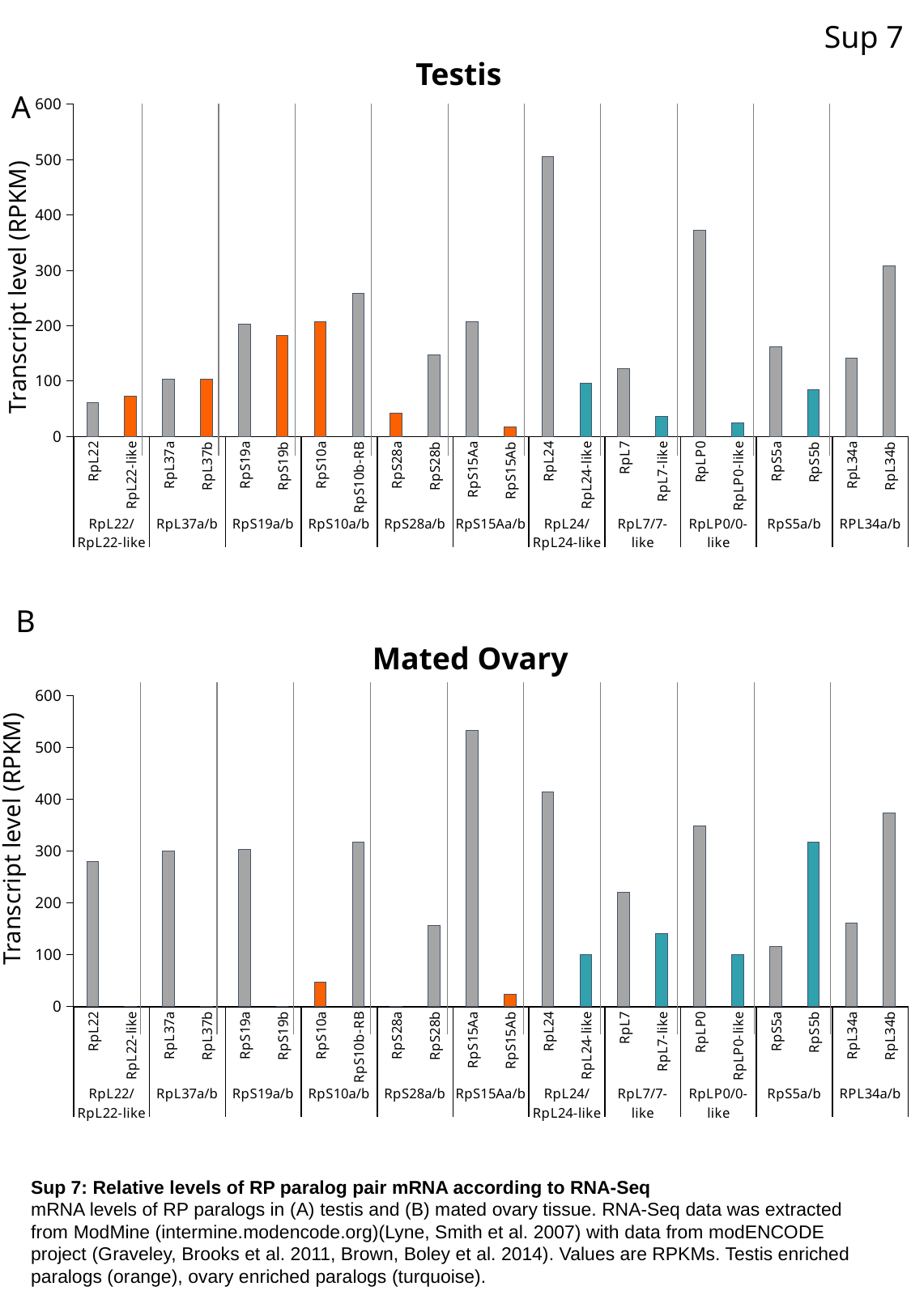

Sup 7
Testis
A
### Chart
| Category | Testis |
|---|---|
| RpL22 | 61.0 |
| RpL22-like | 73.0 |
| RpL37a | 104.0 |
| RpL37b | 103.0 |
| RpS19a | 203.0 |
| RpS19b | 182.0 |
| RpS10a | 207.0 |
| RpS10b-RB | 259.0 |
| RpS28a | 42.0 |
| RpS28b | 148.0 |
| RpS15Aa | 207.0 |
| RpS15Ab | 17.0 |
| RpL24 | 505.0 |
| RpL24-like | 96.0 |
| RpL7 | 123.0 |
| RpL7-like | 37.0 |
| RpLP0 | 373.0 |
| RpLP0-like | 24.0 |
| RpS5a | 162.0 |
| RpS5b | 84.0 |
| RpL34a | 142.0 |
| RpL34b | 308.0 |Transcript level (RPKM)
B
Mated Ovary
### Chart
| Category | Mated Ovary |
|---|---|
| RpL22 | 280.0 |
| RpL22-like | 1.0 |
| RpL37a | 300.0 |
| RpL37b | 0.0 |
| RpS19a | 304.0 |
| RpS19b | 0.0 |
| RpS10a | 47.0 |
| RpS10b-RB | 317.0 |
| RpS28a | 0.0 |
| RpS28b | 156.0 |
| RpS15Aa | 533.0 |
| RpS15Ab | 24.0 |
| RpL24 | 414.0 |
| RpL24-like | 101.0 |
| RpL7 | 220.0 |
| RpL7-like | 141.0 |
| RpLP0 | 349.0 |
| RpLP0-like | 101.0 |
| RpS5a | 116.0 |
| RpS5b | 318.0 |
| RpL34a | 161.0 |
| RpL34b | 374.0 |Transcript level (RPKM)
Sup 7: Relative levels of RP paralog pair mRNA according to RNA-Seq
mRNA levels of RP paralogs in (A) testis and (B) mated ovary tissue. RNA-Seq data was extracted from ModMine (intermine.modencode.org)(Lyne, Smith et al. 2007) with data from modENCODE project (Graveley, Brooks et al. 2011, Brown, Boley et al. 2014). Values are RPKMs. Testis enriched paralogs (orange), ovary enriched paralogs (turquoise).

## Slide 2
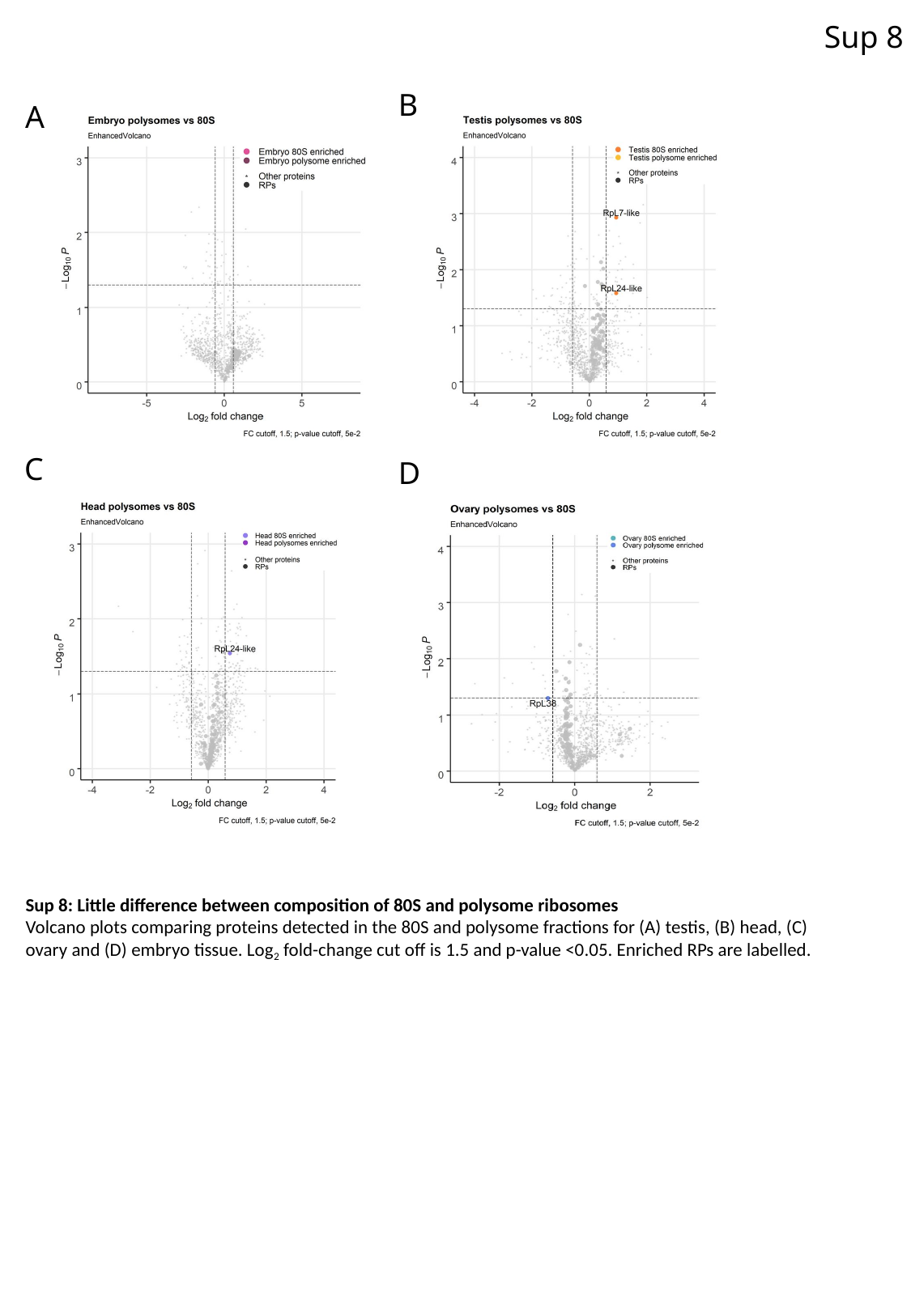

Sup 8
B
A
C
D
Sup 8: Little difference between composition of 80S and polysome ribosomes
Volcano plots comparing proteins detected in the 80S and polysome fractions for (A) testis, (B) head, (C) ovary and (D) embryo tissue. Log2 fold-change cut off is 1.5 and p-value <0.05. Enriched RPs are labelled.
