# Supplementary material for: Ribosome heterogeneity in Drosophila melanogaster gonads through paralog-switching
Source: Nucleic Acids Res. 2021 Jul 20;50(4):2240–57. doi: 10.1093/nar/gkab606 (PMC8887423; doi:10.1093/nar/gkab606)
Supplement: gkab606_Supplemental_Files [file gkab606_supplemental_files.zip › Sup12_15.pptx]

## Slide 1
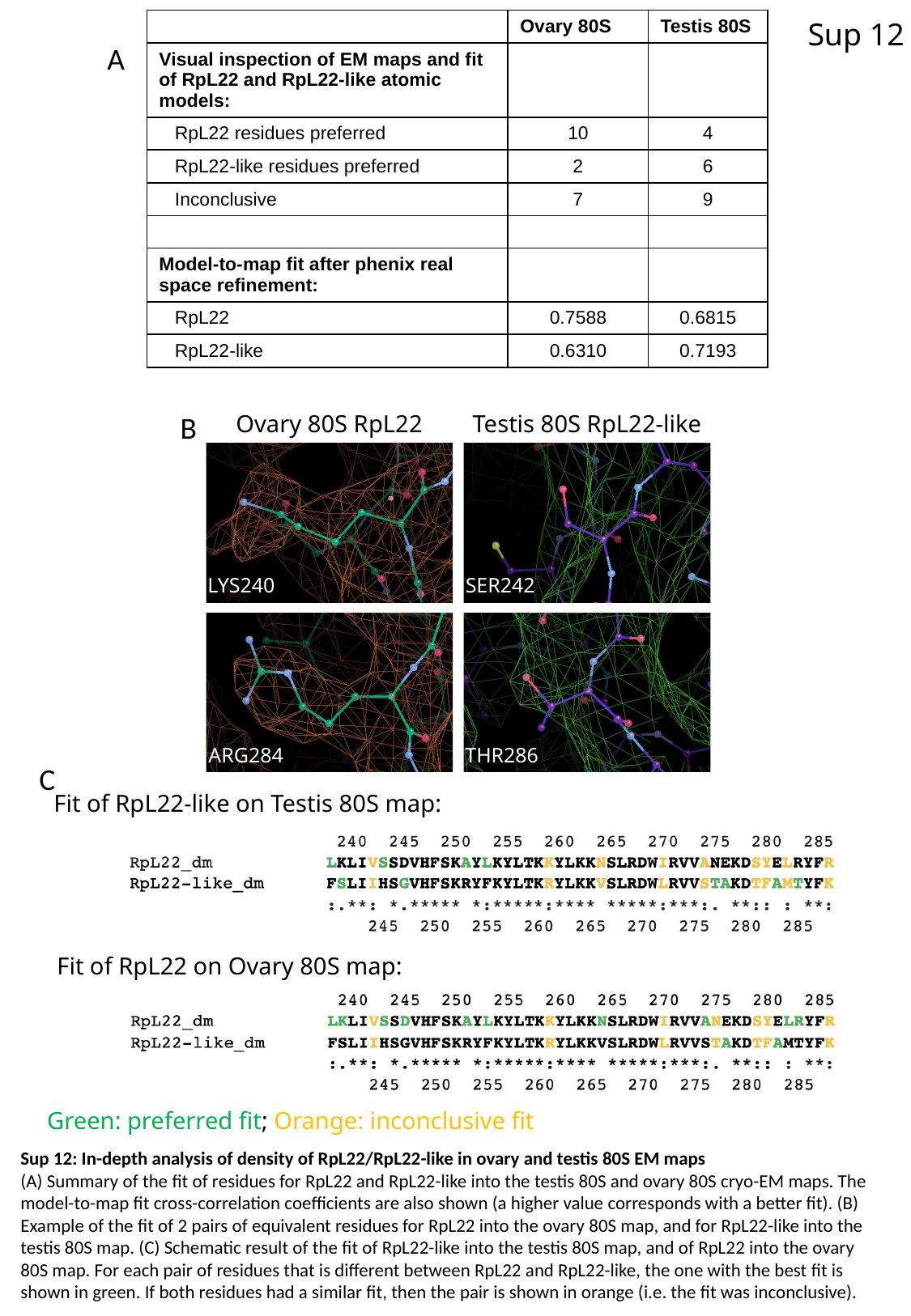

Sup 12
| | Ovary 80S | Testis 80S |
| --- | --- | --- |
| Visual inspection of EM maps and fit of RpL22 and RpL22-like atomic models: | | |
| RpL22 residues preferred | 10 | 4 |
| RpL22-like residues preferred | 2 | 6 |
| Inconclusive | 7 | 9 |
| | | |
| Model-to-map fit after phenix real space refinement: | | |
| RpL22 | 0.7588 | 0.6815 |
| RpL22-like | 0.6310 | 0.7193 |
A
B
Ovary 80S RpL22
Testis 80S RpL22-like
LYS240
SER242
THR286
ARG284
C
Fit of RpL22-like on Testis 80S map:
Fit of RpL22 on Ovary 80S map:
Green: preferred fit; Orange: inconclusive fit
Sup 12: In-depth analysis of density of RpL22/RpL22-like in ovary and testis 80S EM maps
(A) Summary of the fit of residues for RpL22 and RpL22-like into the testis 80S and ovary 80S cryo-EM maps. The model-to-map fit cross-correlation coefficients are also shown (a higher value corresponds with a better fit). (B) Example of the fit of 2 pairs of equivalent residues for RpL22 into the ovary 80S map, and for RpL22-like into the testis 80S map. (C) Schematic result of the fit of RpL22-like into the testis 80S map, and of RpL22 into the ovary 80S map. For each pair of residues that is different between RpL22 and RpL22-like, the one with the best fit is shown in green. If both residues had a similar fit, then the pair is shown in orange (i.e. the fit was inconclusive).

## Slide 2
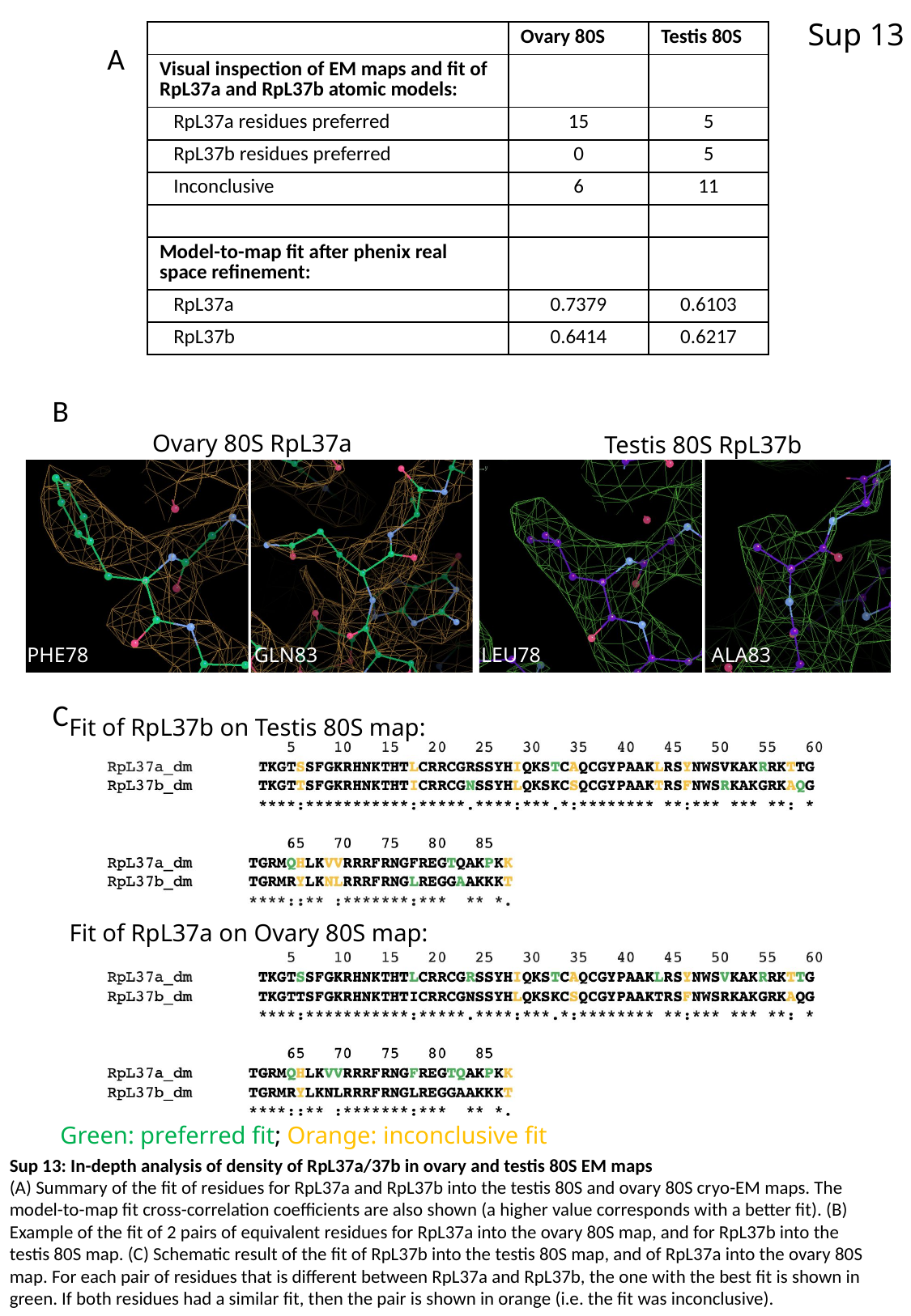

Sup 13
| | Ovary 80S | Testis 80S |
| --- | --- | --- |
| Visual inspection of EM maps and fit of RpL37a and RpL37b atomic models: | | |
| RpL37a residues preferred | 15 | 5 |
| RpL37b residues preferred | 0 | 5 |
| Inconclusive | 6 | 11 |
| | | |
| Model-to-map fit after phenix real space refinement: | | |
| RpL37a | 0.7379 | 0.6103 |
| RpL37b | 0.6414 | 0.6217 |
A
B
Ovary 80S RpL37a
Testis 80S RpL37b
PHE78
GLN83
LEU78
ALA83
C
Fit of RpL37b on Testis 80S map:
Fit of RpL37a on Ovary 80S map:
Green: preferred fit; Orange: inconclusive fit
Sup 13: In-depth analysis of density of RpL37a/37b in ovary and testis 80S EM maps
(A) Summary of the fit of residues for RpL37a and RpL37b into the testis 80S and ovary 80S cryo-EM maps. The model-to-map fit cross-correlation coefficients are also shown (a higher value corresponds with a better fit). (B) Example of the fit of 2 pairs of equivalent residues for RpL37a into the ovary 80S map, and for RpL37b into the testis 80S map. (C) Schematic result of the fit of RpL37b into the testis 80S map, and of RpL37a into the ovary 80S map. For each pair of residues that is different between RpL37a and RpL37b, the one with the best fit is shown in green. If both residues had a similar fit, then the pair is shown in orange (i.e. the fit was inconclusive).

## Slide 3
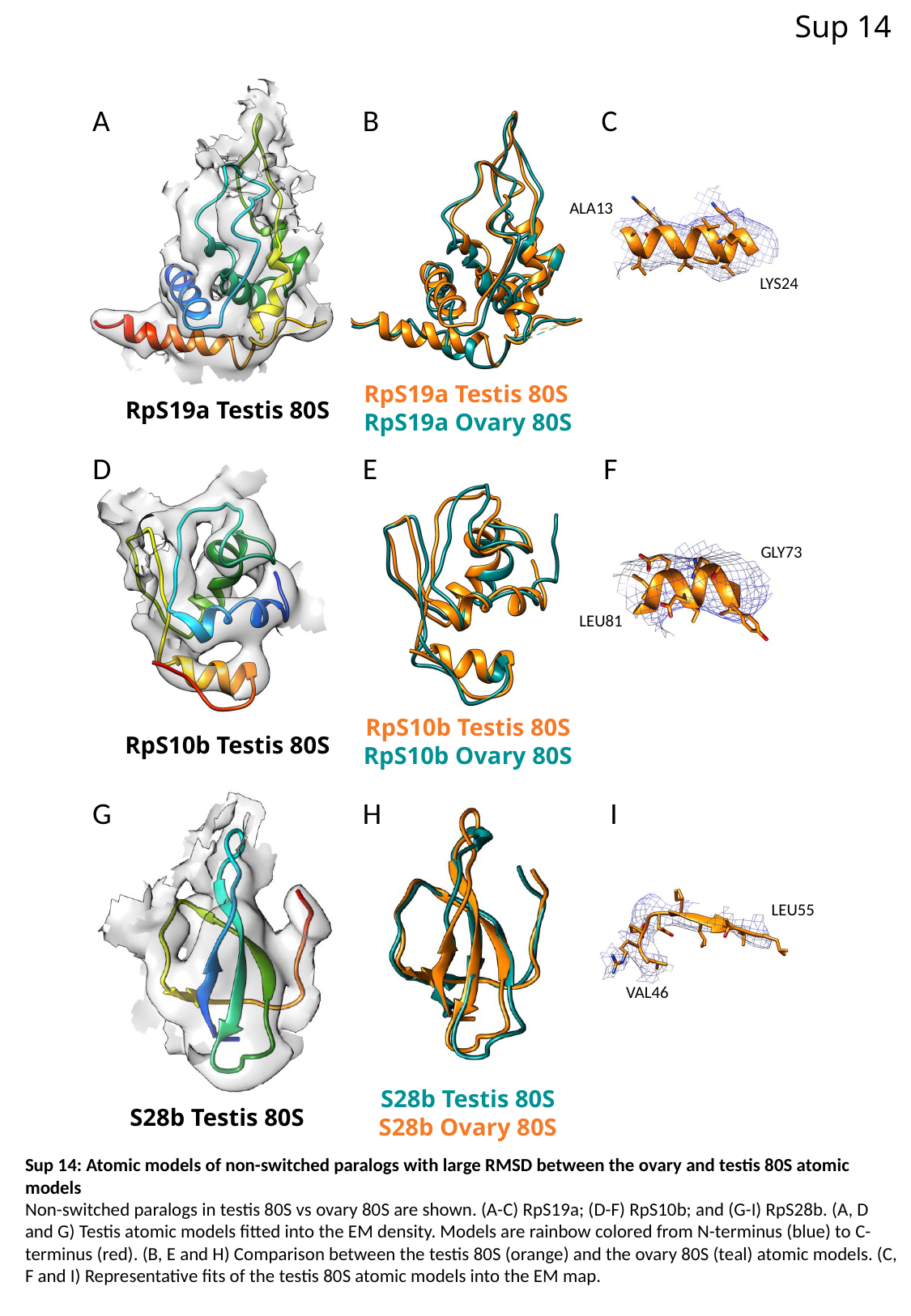

Sup 14
A
B
C
ALA13
LYS24
RpS19a Testis 80S
RpS19a Ovary 80S
RpS19a Testis 80S
D
E
F
GLY73
LEU81
RpS10b Testis 80S
RpS10b Ovary 80S
RpS10b Testis 80S
G
H
I
LEU55
VAL46
S28b Testis 80S
S28b Ovary 80S
S28b Testis 80S
Sup 14: Atomic models of non-switched paralogs with large RMSD between the ovary and testis 80S atomic models
Non-switched paralogs in testis 80S vs ovary 80S are shown. (A-C) RpS19a; (D-F) RpS10b; and (G-I) RpS28b. (A, D and G) Testis atomic models fitted into the EM density. Models are rainbow colored from N-terminus (blue) to C-terminus (red). (B, E and H) Comparison between the testis 80S (orange) and the ovary 80S (teal) atomic models. (C, F and I) Representative fits of the testis 80S atomic models into the EM map.

## Slide 4
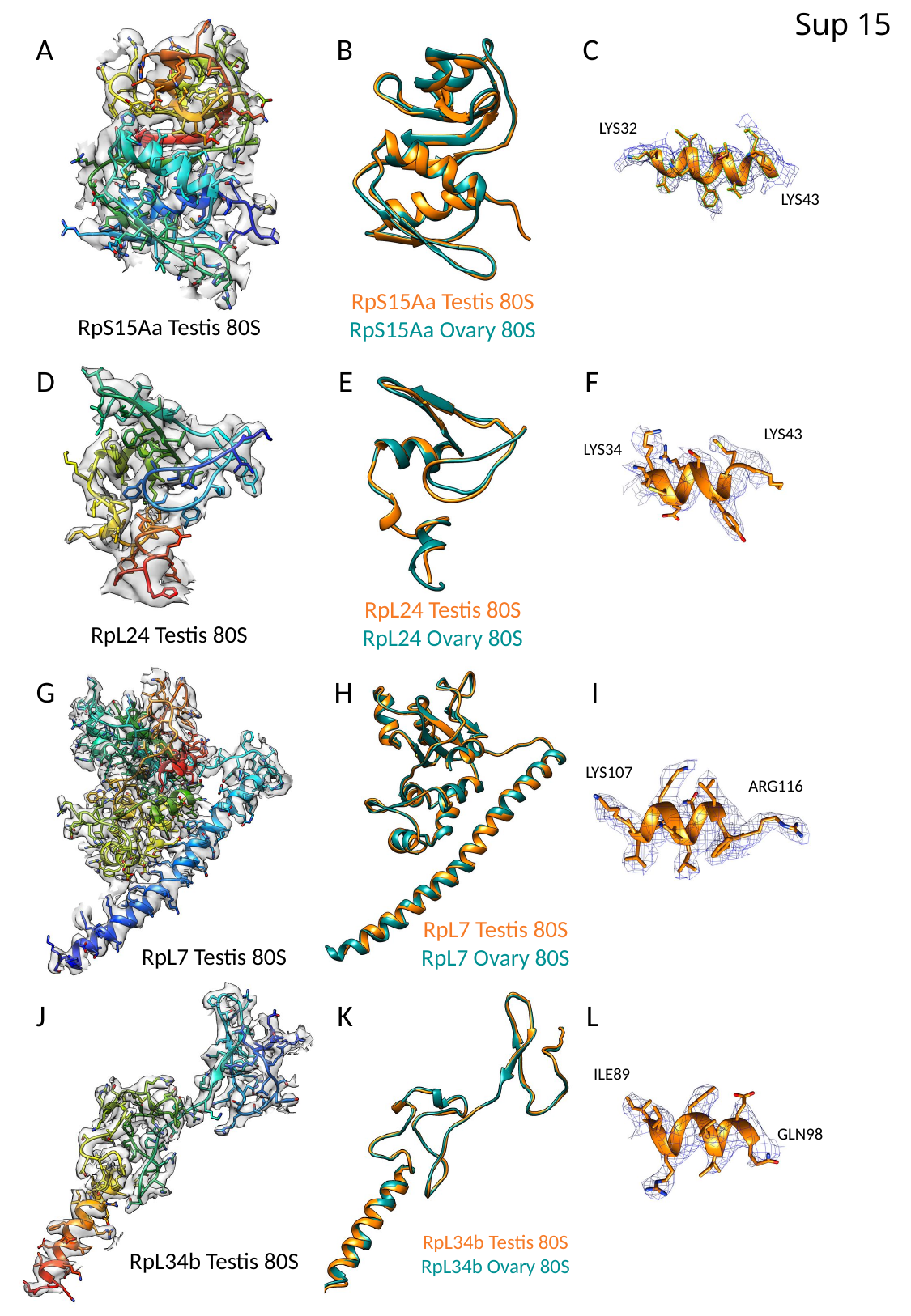

Sup 15
A
B
C
LYS32
LYS43
RpS15Aa Testis 80S
RpS15Aa Ovary 80S
RpS15Aa Testis 80S
D
E
F
LYS43
LYS34
RpL24 Testis 80S
RpL24 Ovary 80S
RpL24 Testis 80S
G
H
I
LYS107
ARG116
RpL7 Testis 80S
RpL7 Ovary 80S
RpL7 Testis 80S
J
K
L
ILE89
GLN98
RpL34b Testis 80S
RpL34b Ovary 80S
RpL34b Testis 80S

## Slide 5
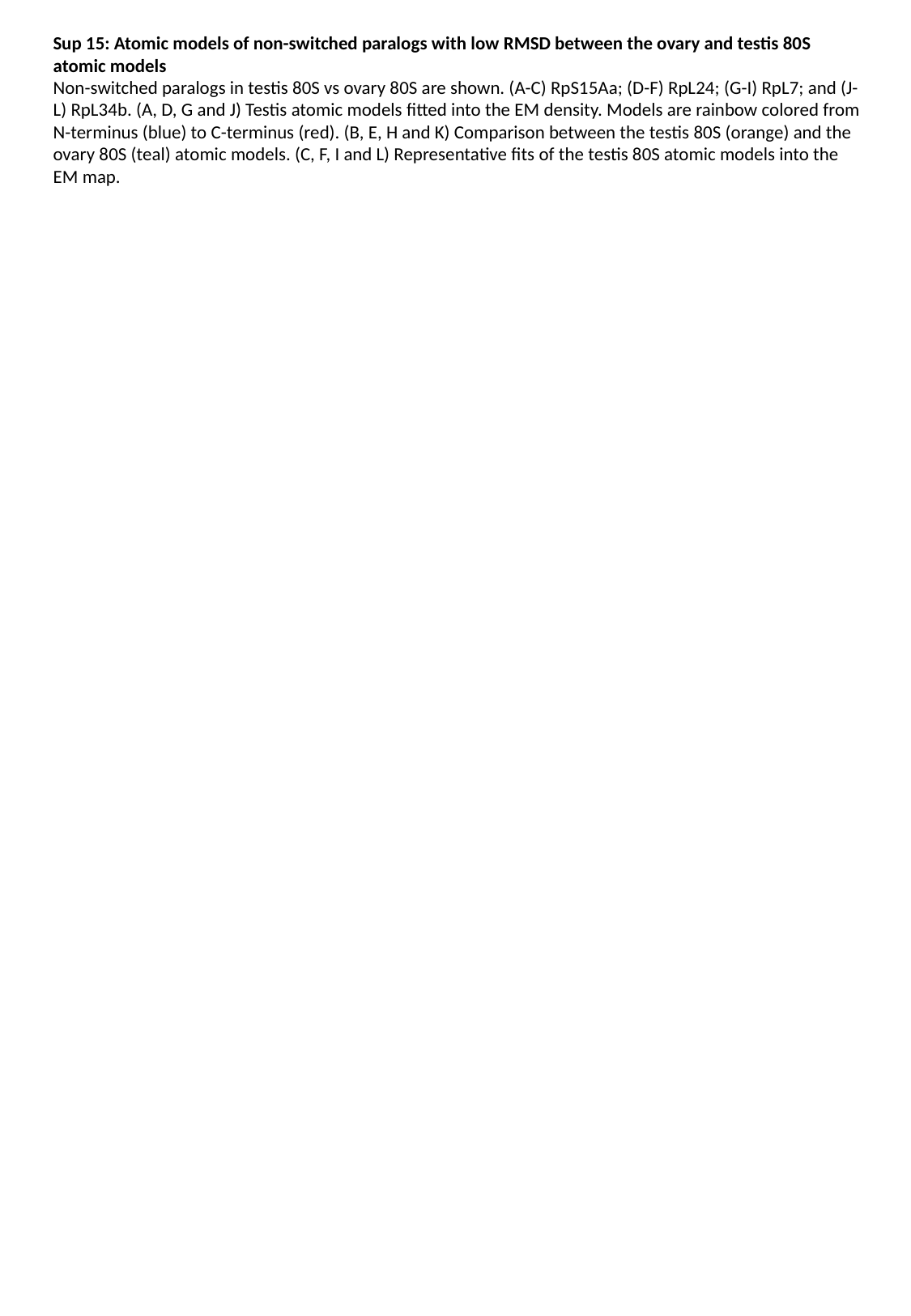

Sup 15: Atomic models of non-switched paralogs with low RMSD between the ovary and testis 80S atomic models
Non-switched paralogs in testis 80S vs ovary 80S are shown. (A-C) RpS15Aa; (D-F) RpL24; (G-I) RpL7; and (J-L) RpL34b. (A, D, G and J) Testis atomic models fitted into the EM density. Models are rainbow colored from N-terminus (blue) to C-terminus (red). (B, E, H and K) Comparison between the testis 80S (orange) and the ovary 80S (teal) atomic models. (C, F, I and L) Representative fits of the testis 80S atomic models into the EM map.
