# Supplementary material for: Ribosome heterogeneity in Drosophila melanogaster gonads through paralog-switching
Source: Nucleic Acids Res. 2021 Jul 20;50(4):2240–57. doi: 10.1093/nar/gkab606 (PMC8887423; doi:10.1093/nar/gkab606)
Supplement: gkab606_Supplemental_Files [file gkab606_supplemental_files.zip › Sup16.pptx]

## Slide 1
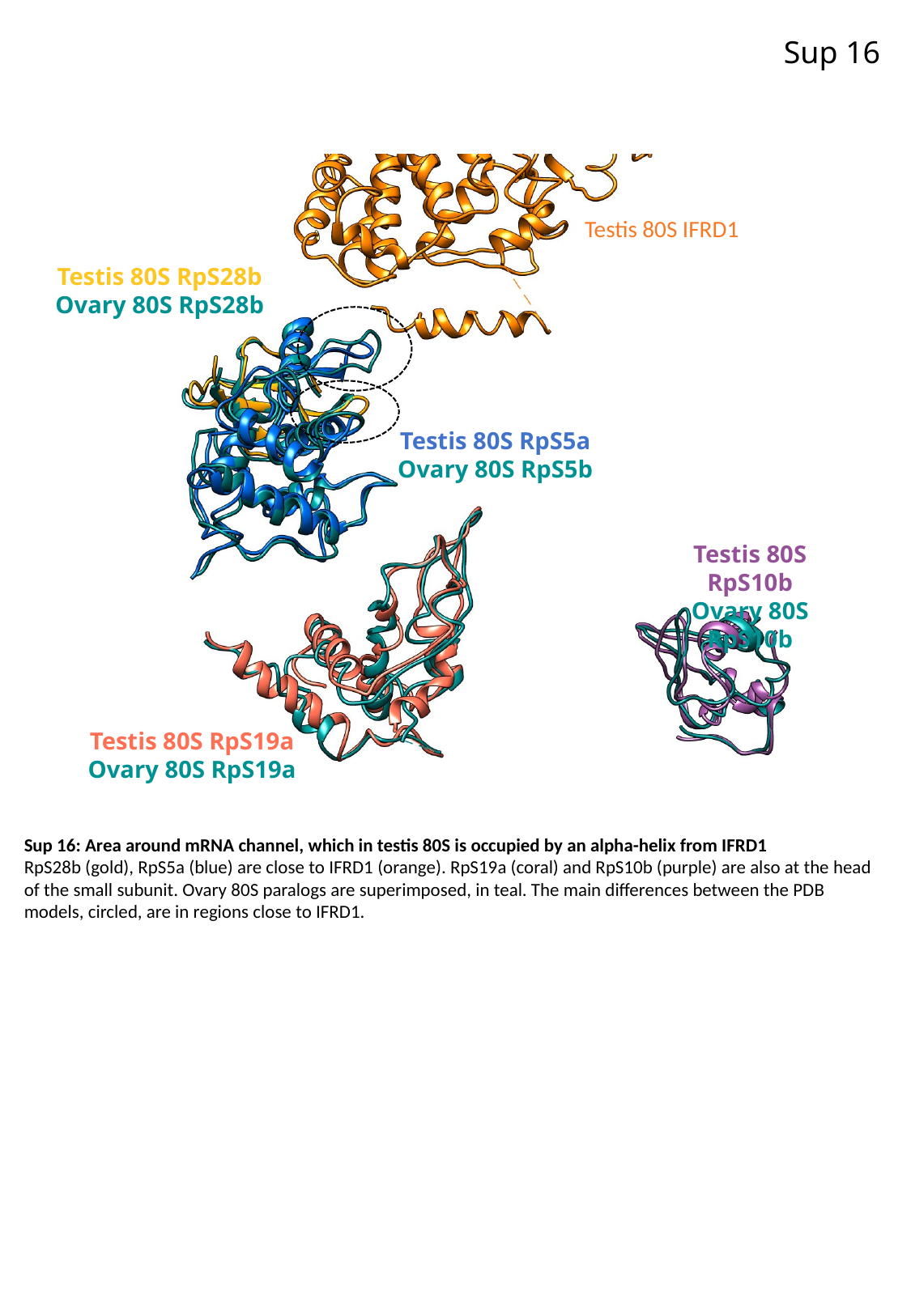

Sup 16
Testis 80S IFRD1
Testis 80S RpS28b
Ovary 80S RpS28b
Testis 80S RpS5a
Ovary 80S RpS5b
Testis 80S RpS10b
Ovary 80S RpS10b
Testis 80S RpS19a
Ovary 80S RpS19a
Sup 16: Area around mRNA channel, which in testis 80S is occupied by an alpha-helix from IFRD1
RpS28b (gold), RpS5a (blue) are close to IFRD1 (orange). RpS19a (coral) and RpS10b (purple) are also at the head of the small subunit. Ovary 80S paralogs are superimposed, in teal. The main differences between the PDB models, circled, are in regions close to IFRD1.
