# Supplementary material for: Ribosome heterogeneity in Drosophila melanogaster gonads through paralog-switching
Source: Nucleic Acids Res. 2021 Jul 20;50(4):2240–57. doi: 10.1093/nar/gkab606 (PMC8887423; doi:10.1093/nar/gkab606)
Supplement: gkab606_Supplemental_Files [file gkab606_supplemental_files.zip › Sup17.pptx]

## Slide 1
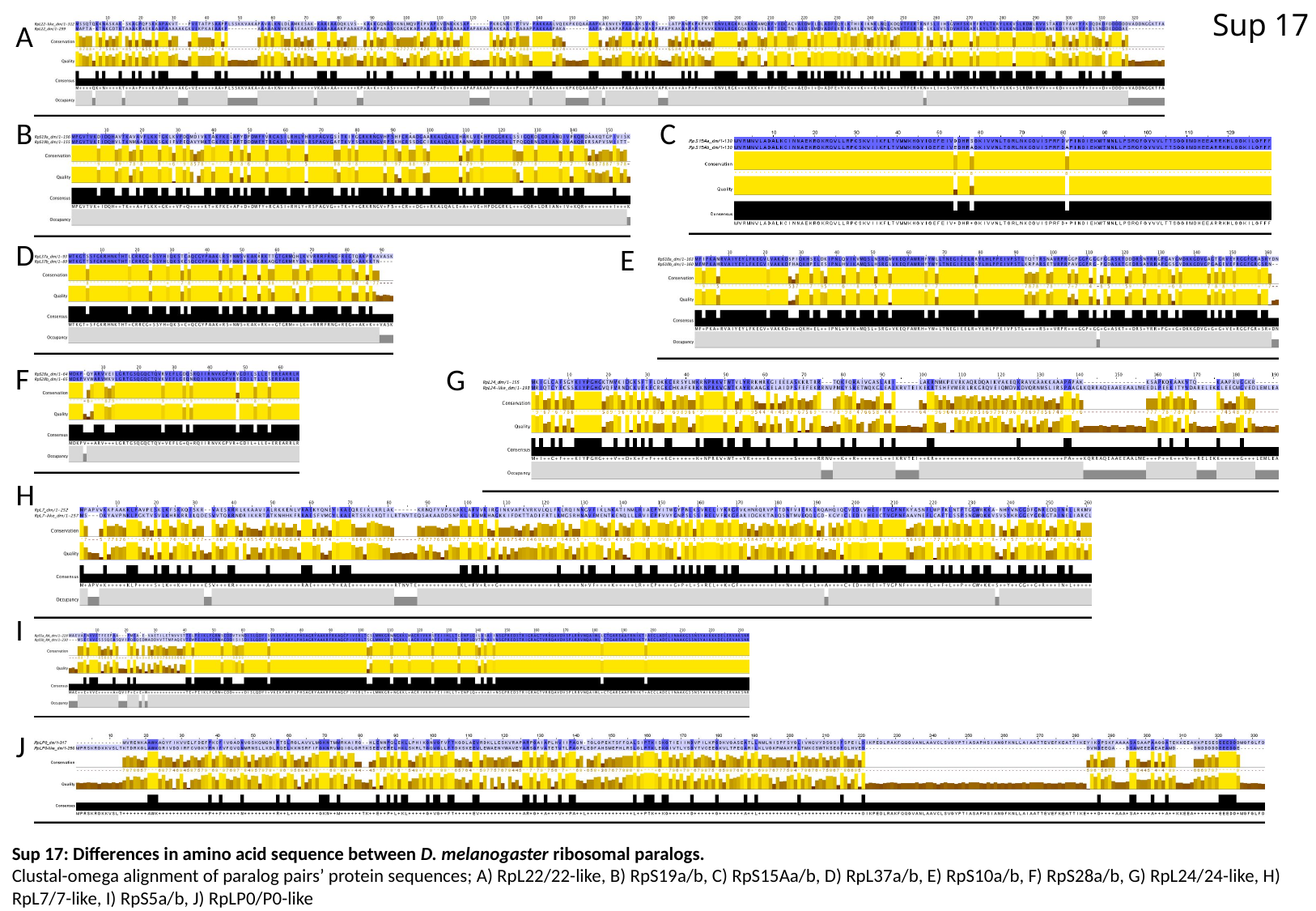

Sup 17
A
B
C
D
E
F
G
H
I
J
Sup 17: Differences in amino acid sequence between D. melanogaster ribosomal paralogs.
Clustal-omega alignment of paralog pairs’ protein sequences; A) RpL22/22-like, B) RpS19a/b, C) RpS15Aa/b, D) RpL37a/b, E) RpS10a/b, F) RpS28a/b, G) RpL24/24-like, H) RpL7/7-like, I) RpS5a/b, J) RpLP0/P0-like
