# Supplementary material for: Ribosome heterogeneity in Drosophila melanogaster gonads through paralog-switching
Source: Nucleic Acids Res. 2021 Jul 20;50(4):2240–57. doi: 10.1093/nar/gkab606 (PMC8887423; doi:10.1093/nar/gkab606)
Supplement: gkab606_Supplemental_Files [file gkab606_supplemental_files.zip › Sup5.pptx]

## Slide 1
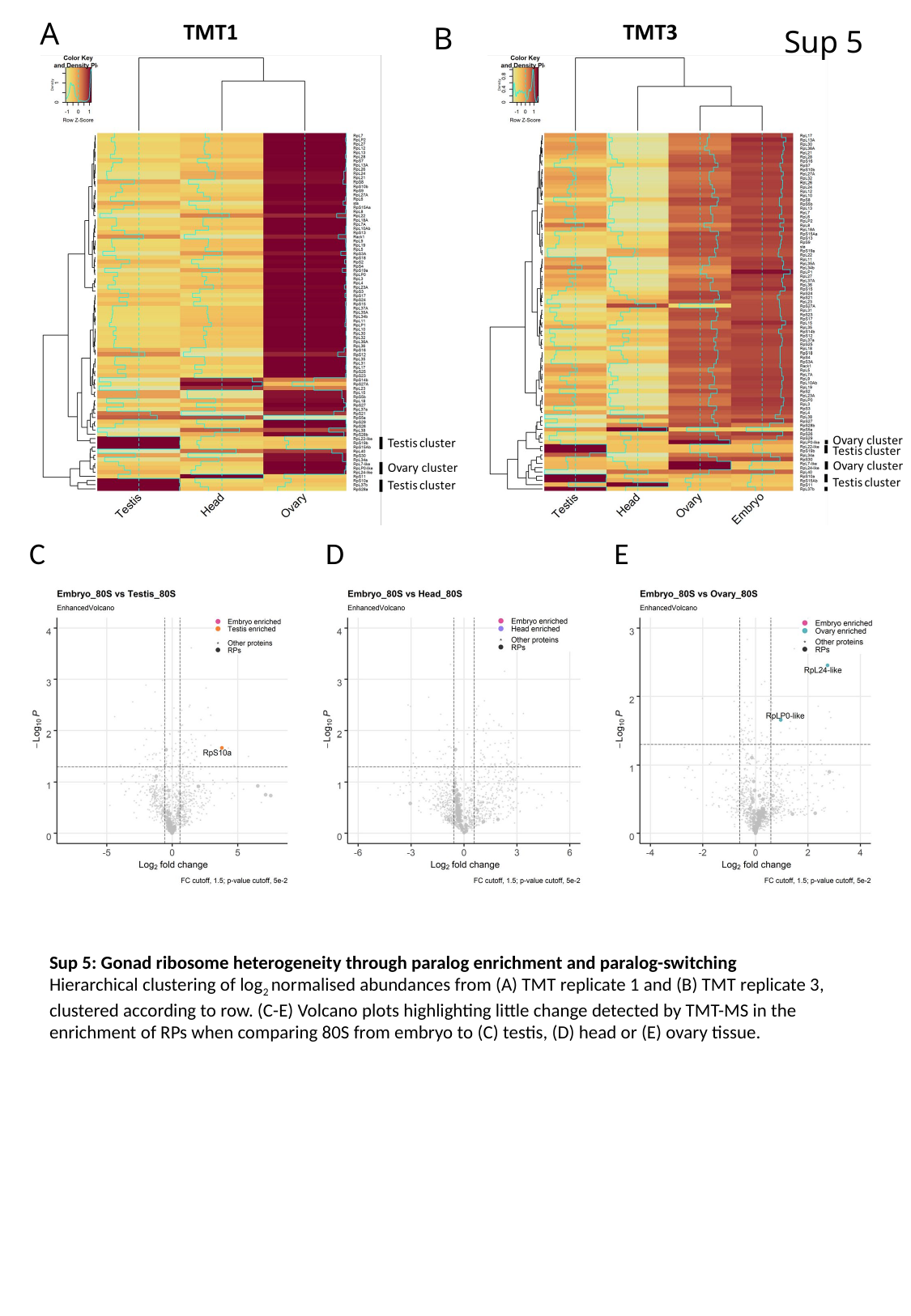

A
B
Sup 5
C
D
E
Sup 5: Gonad ribosome heterogeneity through paralog enrichment and paralog-switching
Hierarchical clustering of log2 normalised abundances from (A) TMT replicate 1 and (B) TMT replicate 3, clustered according to row. (C-E) Volcano plots highlighting little change detected by TMT-MS in the enrichment of RPs when comparing 80S from embryo to (C) testis, (D) head or (E) ovary tissue.
