# Supplementary material for: Ribosome heterogeneity in Drosophila melanogaster gonads through paralog-switching
Source: Nucleic Acids Res. 2021 Jul 20;50(4):2240–57. doi: 10.1093/nar/gkab606 (PMC8887423; doi:10.1093/nar/gkab606)
Supplement: gkab606_Supplemental_Files [file gkab606_supplemental_files.zip › Sup6.pptx]

## Slide 1
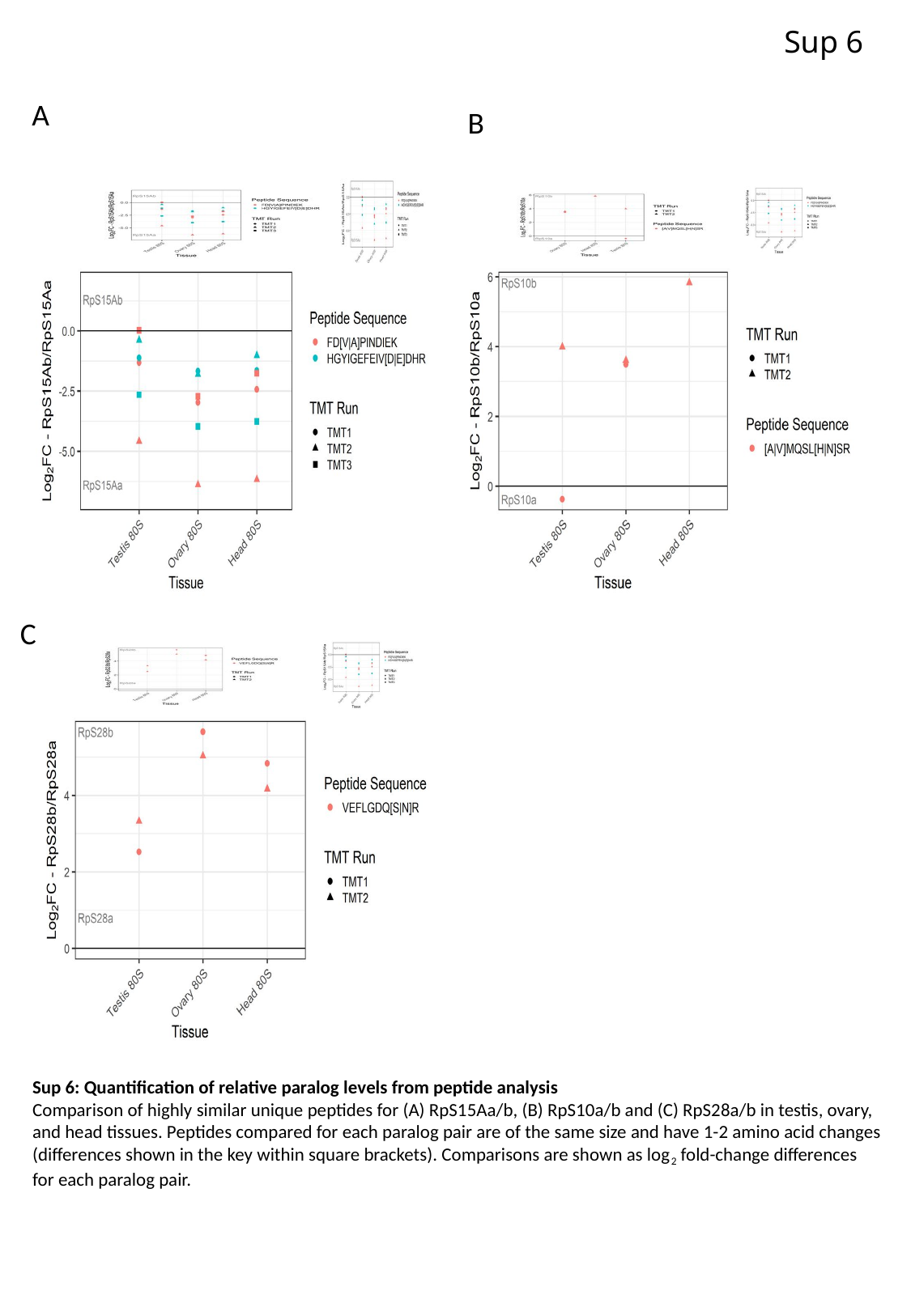

Sup 6
A
B
C
Sup 6: Quantification of relative paralog levels from peptide analysis
Comparison of highly similar unique peptides for (A) RpS15Aa/b, (B) RpS10a/b and (C) RpS28a/b in testis, ovary, and head tissues. Peptides compared for each paralog pair are of the same size and have 1-2 amino acid changes (differences shown in the key within square brackets). Comparisons are shown as log2 fold-change differences for each paralog pair.
