# Supplementary material for: Ribosome heterogeneity in Drosophila melanogaster gonads through paralog-switching
Source: Nucleic Acids Res. 2021 Jul 20;50(4):2240–57. doi: 10.1093/nar/gkab606 (PMC8887423; doi:10.1093/nar/gkab606)
Supplement: gkab606_Supplemental_Files [file gkab606_supplemental_files.zip › Sup9.pptx]

## Slide 1
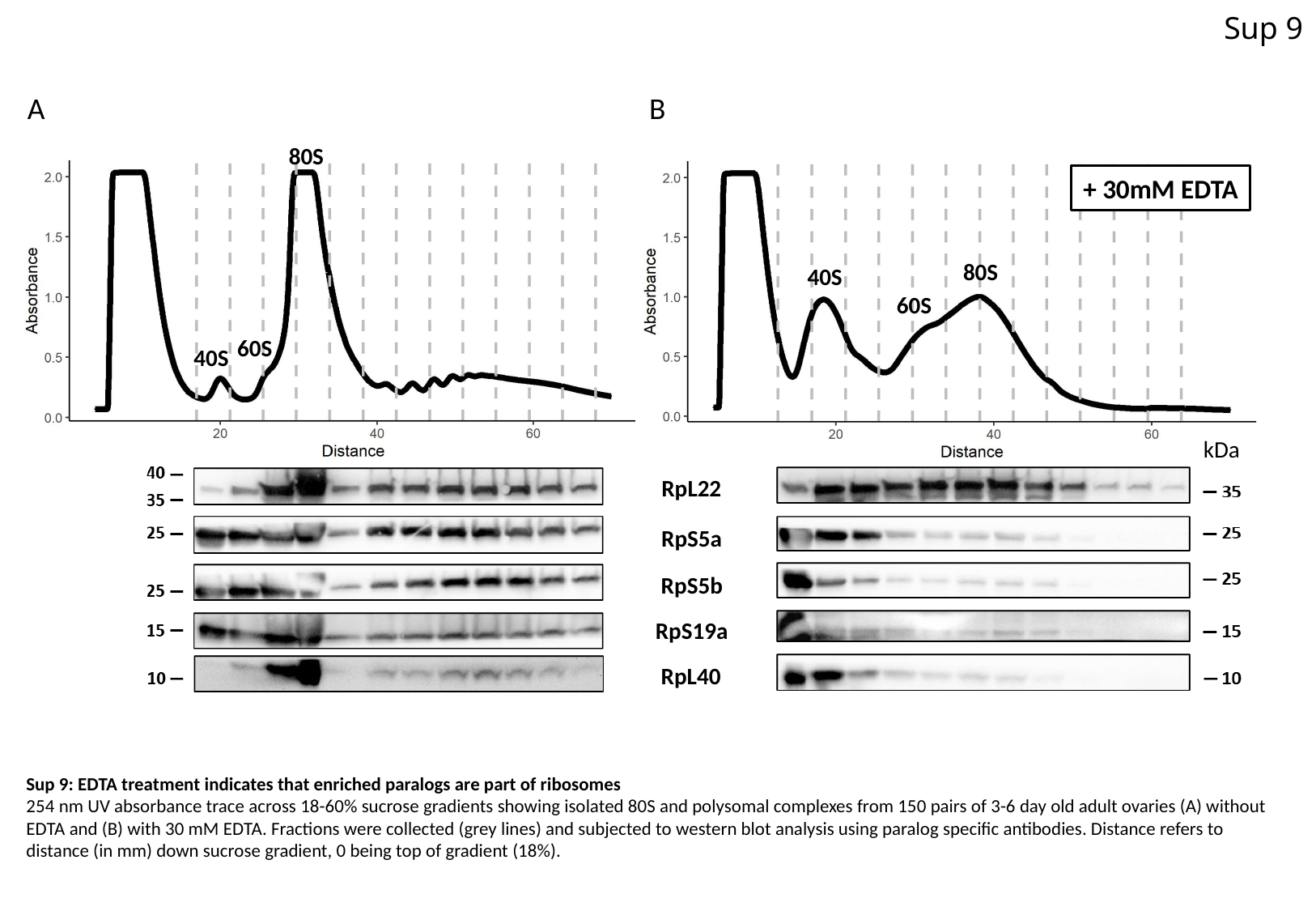

Sup 9
A
B
80S
RpL22
RpS5a
RpS5b
RpS19a
RpL40
+ 30mM EDTA
80S
40S
60S
60S
40S
kDa
Sup 9: EDTA treatment indicates that enriched paralogs are part of ribosomes
254 nm UV absorbance trace across 18-60% sucrose gradients showing isolated 80S and polysomal complexes from 150 pairs of 3-6 day old adult ovaries (A) without EDTA and (B) with 30 mM EDTA. Fractions were collected (grey lines) and subjected to western blot analysis using paralog specific antibodies. Distance refers to distance (in mm) down sucrose gradient, 0 being top of gradient (18%).
