# Supplementary material for: Ribosome heterogeneity in Drosophila melanogaster gonads through paralog-switching
Source: Nucleic Acids Res. 2021 Jul 20;50(4):2240–57. doi: 10.1093/nar/gkab606 (PMC8887423; doi:10.1093/nar/gkab606)
Supplement: gkab606_Supplemental_Files [file gkab606_supplemental_files.zip › Supplementary_Files.pdf]

Supplementary Data

Sup Table 1 - please see separate SupTable1.xls file:
Processed TMT-MS data including the fold-changes of proteins detected in monosomal and polysomal complexes across testis, ovary, embryo and head tissue.

Sup Table 2

	Testis 80S	Ovary 80S	Testis Polysomes
**Data collection**			
**Microscope**	FEI Titan KRIOS	FEI Titan KRIOS	FEI Titan KRIOS
**Voltage (keV)**	300	300	300
**Detector**	FEI Falcon III	FEI Falcon III	FEI Falcon III
**Magnification**	x75,000	x75,000	x75,000
**Defocus range**	-2 to -4	-2 to -4	-2 to -4
**Pixel size (Å)**	1.065	1.065	1.065
**Electron dose (e^-/Å^2)**	80	80	80
**Electron dose per frame (e^-/Å^2)**	1.35	1.35	1.35
**Exposure (sec)**	2	2	2
**No. of frames**	60	60	60
**No. of micrographs**	5,241	9,076	2,758
**Data processing**			
**Symmetry Point Group**	C1	C1	C1
**Final particle number**	46,878	185,913	10,392
**Map average resolution (Å, 0.143 FSC threshold)**	3.5	3.0	4.9
**Map sharpening B-factor (Å^2)**	-150	-118	-197
**Multi-body refinement**		N/A	N/A
**Large subunit**			
**Map average resolution (Å, 0.143 FSC threshold)**	3.5		
**Map sharpening B-factor (Å^2)**	-143		
**Small subunit without head**			
**Map average resolution (Å, 0.143 FSC threshold)**	3.7		
**Map sharpening B-factor (Å^2)**	-165		
**Head of small subunit**			
**Map average resolution (Å, 0.143 FSC threshold)**	4.8		
**Map sharpening B-factor (Å^2)**	-224		
**Refinement**			
**Initial model (PDB code)**	4v6w	Testis 80S model	Testis 80S model
**Model Composition**			
**Non-hydrogen atoms**	219,765	216,955	219,005
**Amino acid residues**	12,097	11,750	11,764
**Nucleotides**	5,916	5,912	6,003
**R.M.S.D. from ideal geometry**			
**Bond lengths (Å)**	0.020	0.014	0.013
**Bond angles (º)**	1.766	1.484	1.455
**Validation**			
**Clashscore**	12.54	9.16	15.01
**Rotamer outliers (%)**	2.47	1.82	1.43
**Ramachandran plot statistics**			
**Favored (%)**	83.37	82.68	82.92
**Allowed (%)**	15.82	16.32	16.61
**Outliers (%)**	0.81	1.01	0.47
**FSC model – map (Å, 0.5 threshold)**	3.4	3.1	4.6

Sup Table 2: Summary of data collection, image processing, model building, refinement and validation statistics

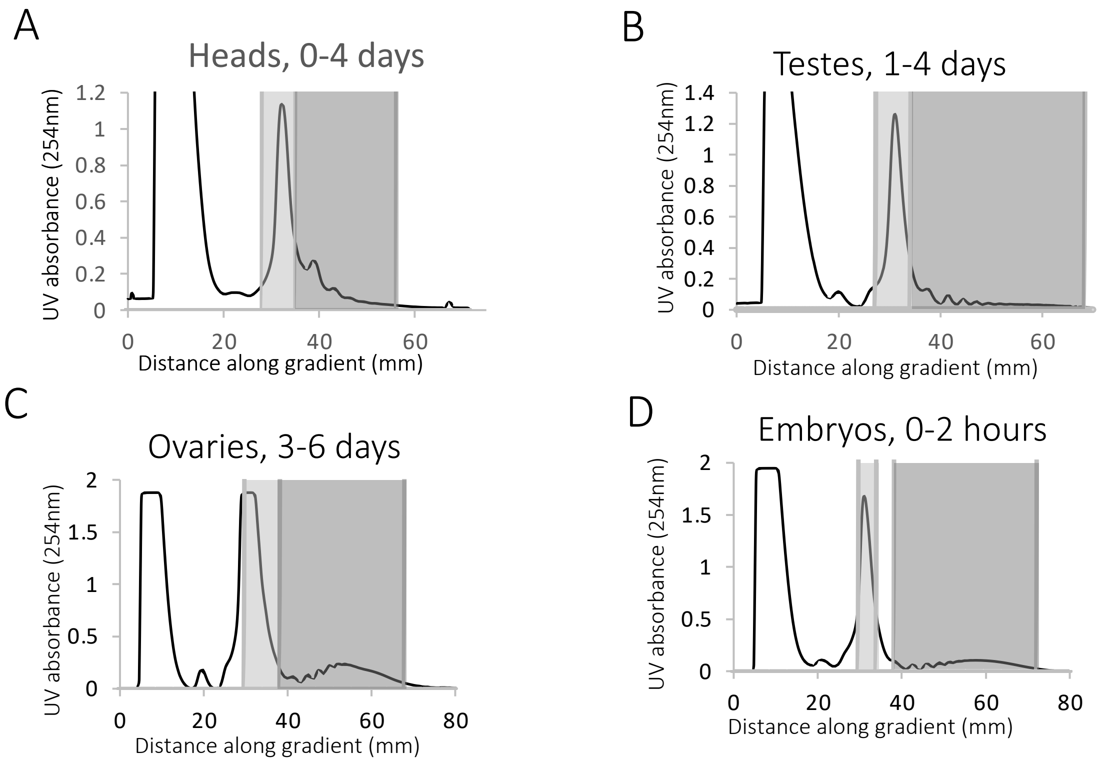

Sup 1: Determined ribosomal composition in tissues and during development
254 nm UV plots across sucrose gradients with 80S and polysomal complexes isolated from (A) 50:50 mixture of female:male 0-3 day old heads. (B) ~500 pairs of 1-4 day old adult testes, (C) ~500 pairs of 3-6 day old adult ovaries, (D) 0-2 hour embryos. Light grey shading indicates fractions used for 80S and dark grey for polysomes.

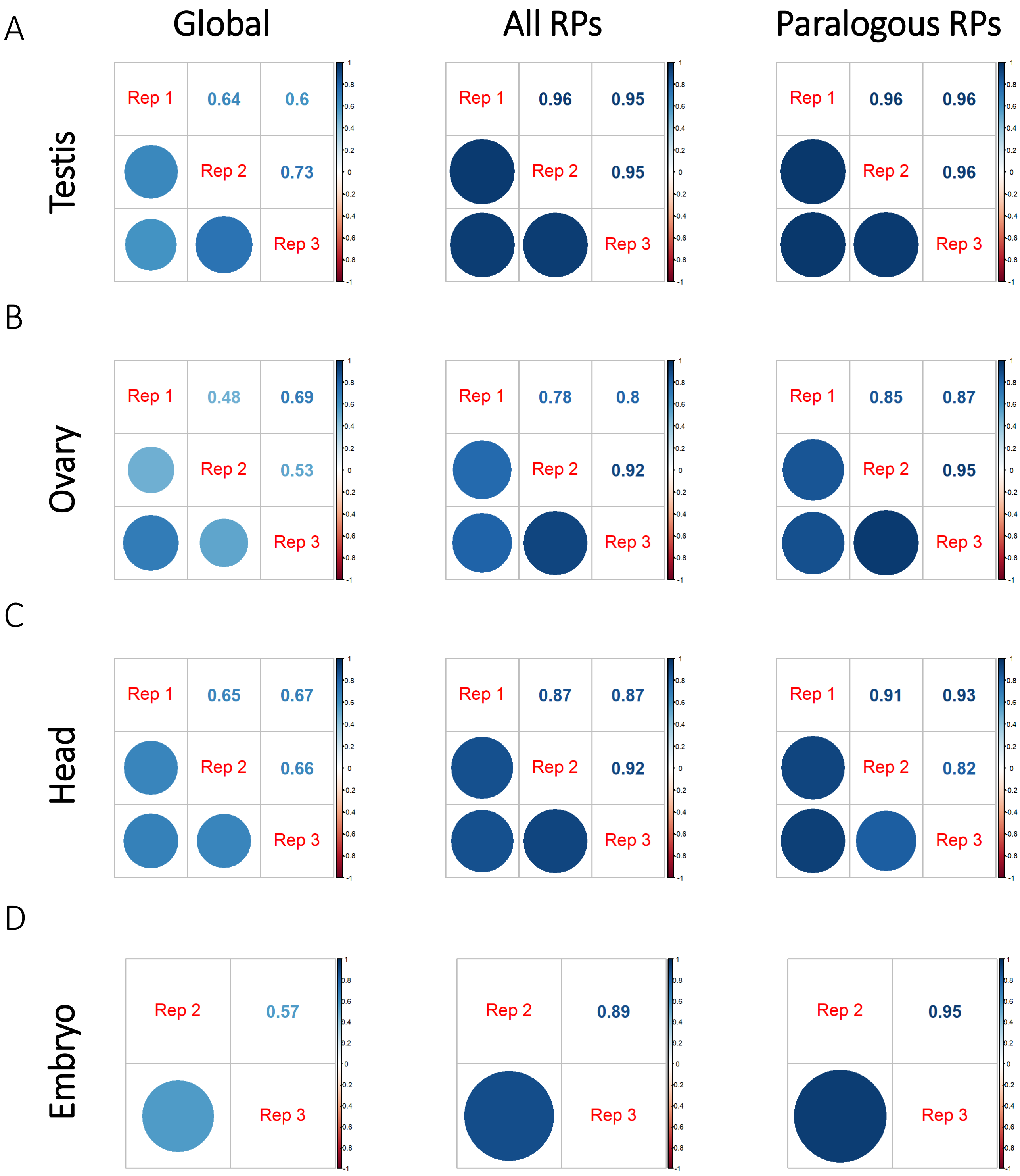

Sup 2: Reproducibility of TMT mass spectrometry experiments
Correlation matrices showing Pearson correlation coefficients when comparing replicates for each tissue. All *Drosophila melanogaster* proteins (global), RPs and paralogous RPs are compared from 80S monosome fractions for (A) testis, (B) ovary, (C) heads and (D) embryo tissue. Size and colour of circles correspond to correlation coefficients for all replicates, which are also stated as numbers. All comparisons were statistically significant (p-value <0.05).

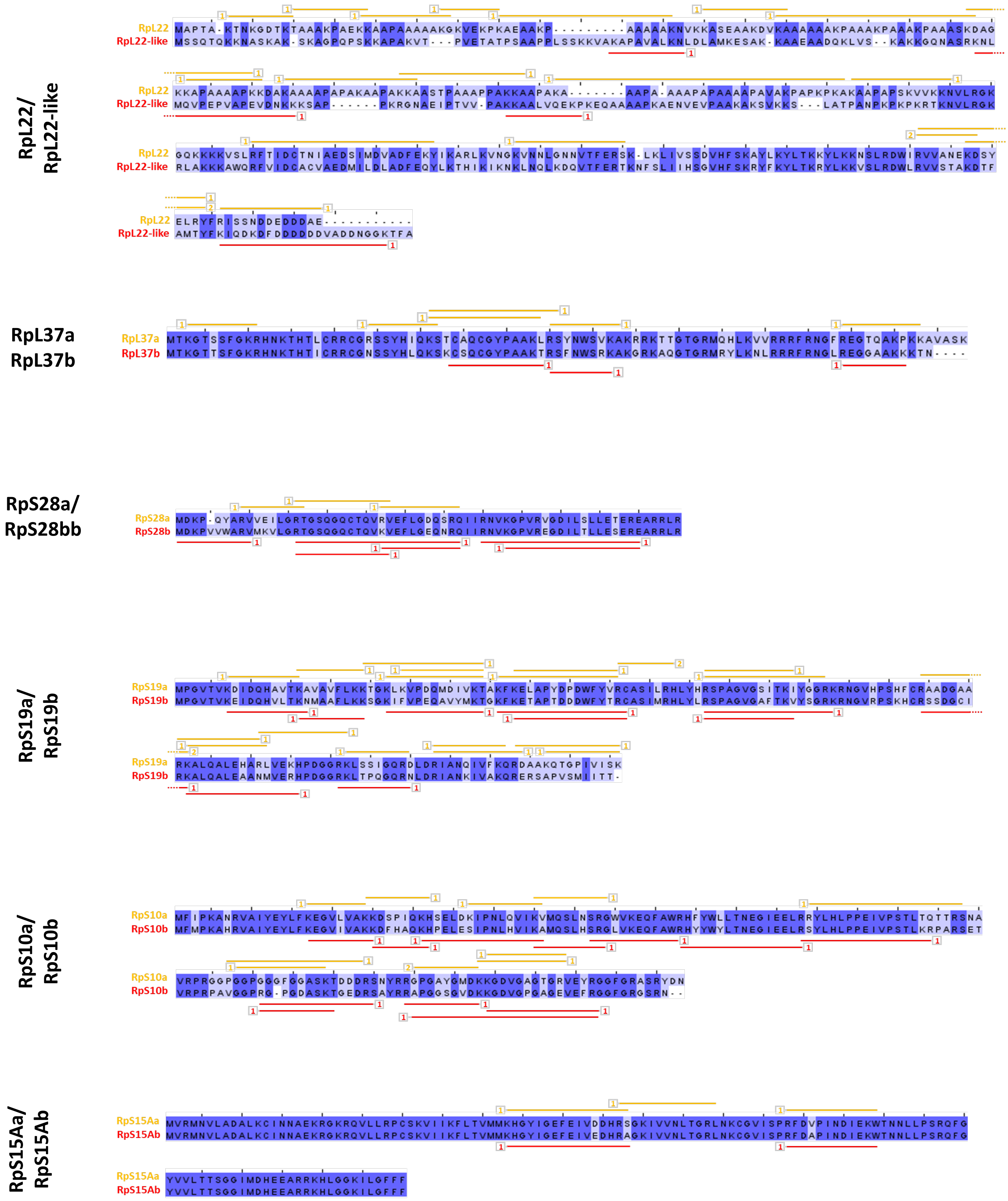

Sup 3 and 4: Unique peptide mapping

Clustal-omega alignment of paralog pairs with unique peptides mapped that was detected during TMT experiments. The confidence of peptide identification is given for each unique peptide: 1 denotes high confidence (<1% false discovery rate, FDR), 2 denotes medium confidence (<5% FDR).

Sup 4

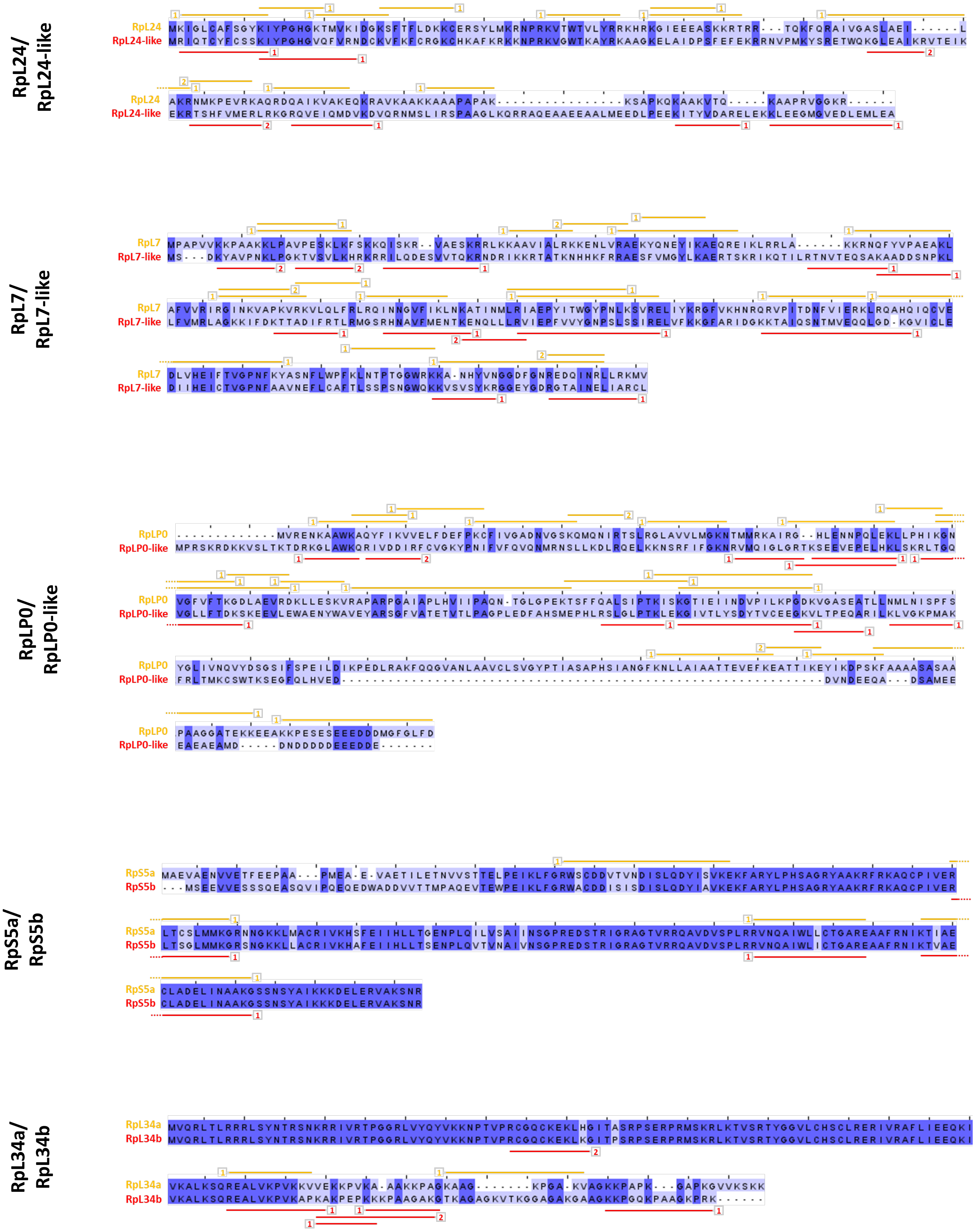
RpL24/ RpL24-like
RpL24
RpL24-like
RpL7/ RpL7-like
RpL7
RpL7-like
RpLP0/ RpLP0-like
RpLP0
RpLP0-like
RpS5a/ RpS5b
RpS5a
RpS5b
RpL34a/ RpL34b
RpL34a
RpL34b

Sup 5

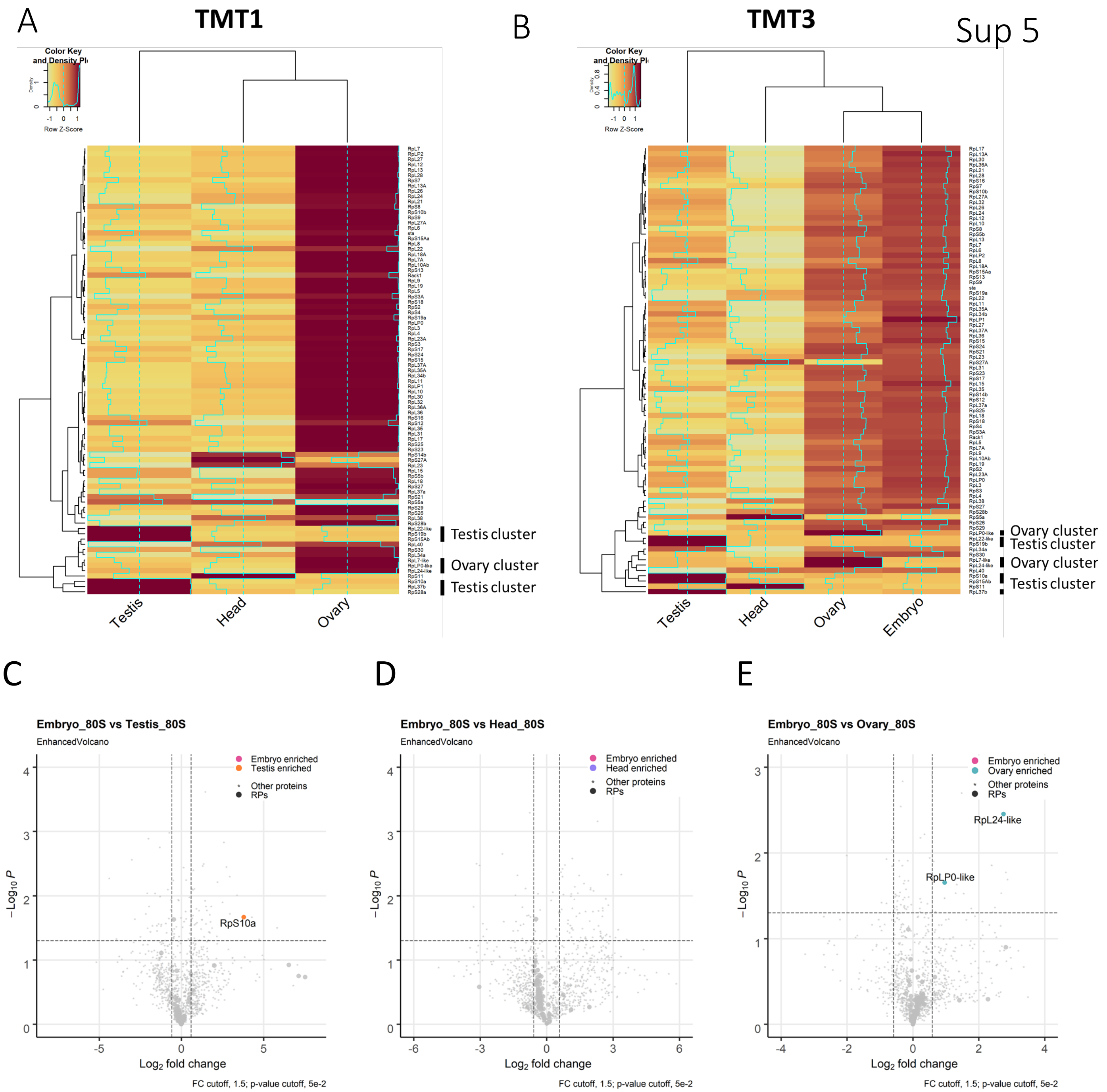

Sup 5: Gonad ribosome heterogeneity through paralog enrichment and paralog-switching
Hierarchical clustering of log_2 normalised abundances from (A) TMT replicate 1 and (B) TMT replicate 3, clustered according to row. (C-E) Volcano plots highlighting little change detected by TMT-MS in the enrichment of RPs when comparing 80S from embryo to (C) testis, (D) head or (E) ovary tissue.

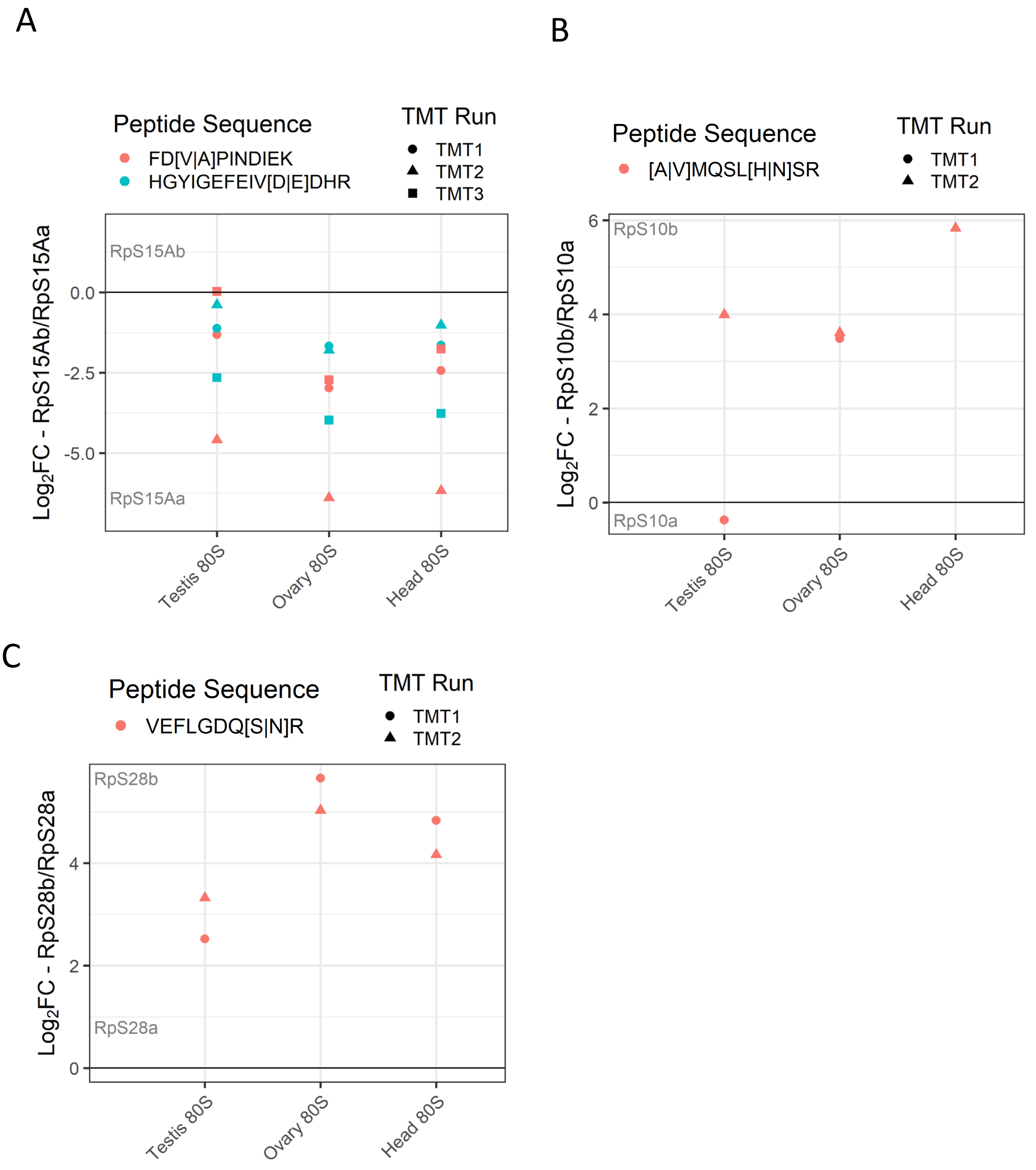

Sup 6: Quantification of relative paralog levels from peptide analysis

Comparison of highly similar unique peptides for (A) RpS15Aa/b, (B) RpS10a/b and (C) RpS28a/b in testis, ovary, and head tissues. Peptides compared for each paralog pair are of the same size and have 1-2 amino acid changes (differences shown in the key within square brackets). Comparisons are shown as log_2 fold-change differences for each paralog pair.

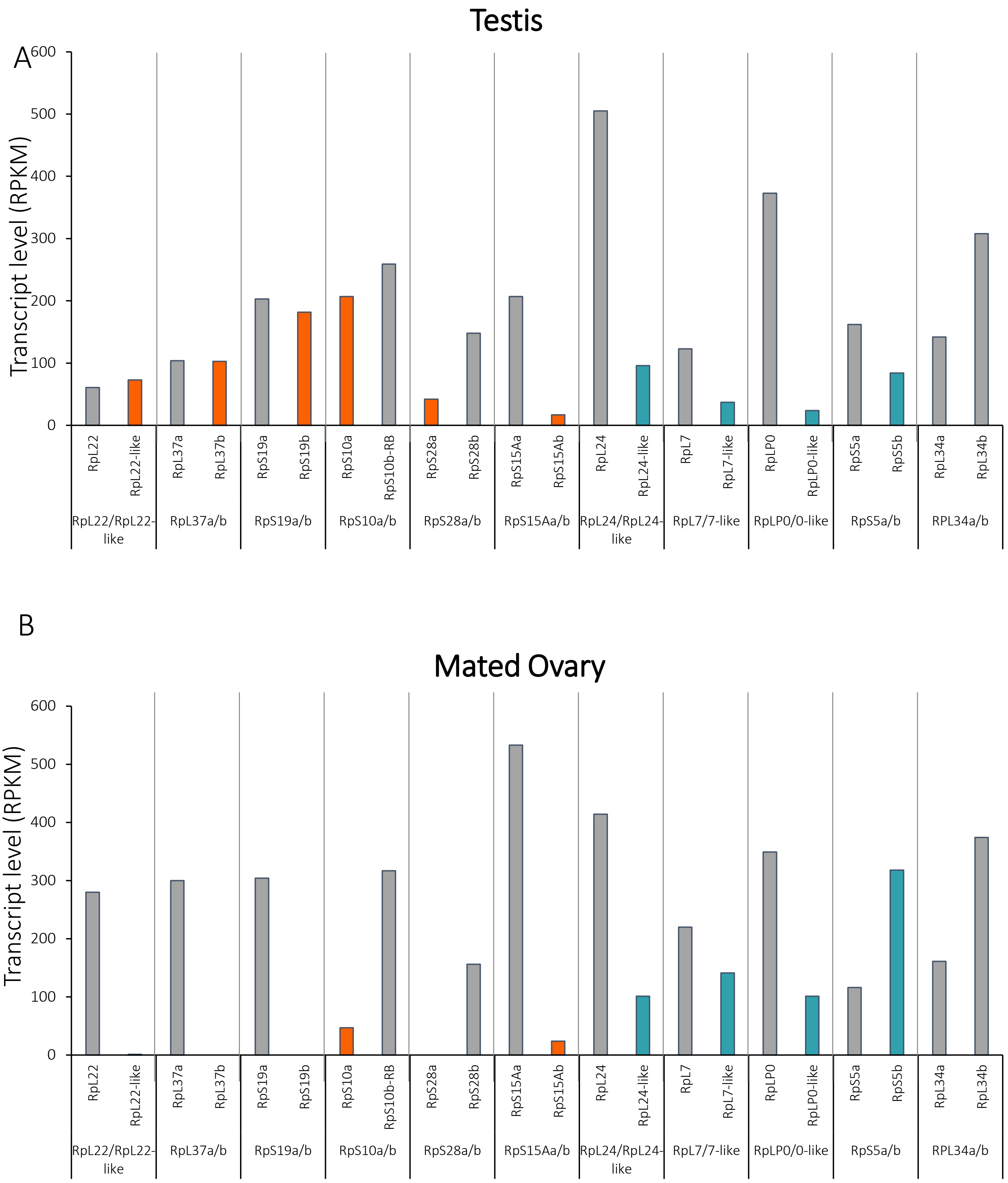

Sup 7: Relative levels of RP paralog pair mRNA according to RNA-Seq
mRNA levels of RP paralogs in (A) testis and (B) mated ovary tissue. RNA-Seq data was extracted from ModMine (intermine.modencode.org)(Lyne, Smith et al. 2007) with data from modENCODE project (Graveley, Brooks et al. 2011, Brown, Boley et al. 2014). Values are RPKMs. Testis enriched paralogs (orange), ovary enriched paralogs (turquoise).

Sup 8

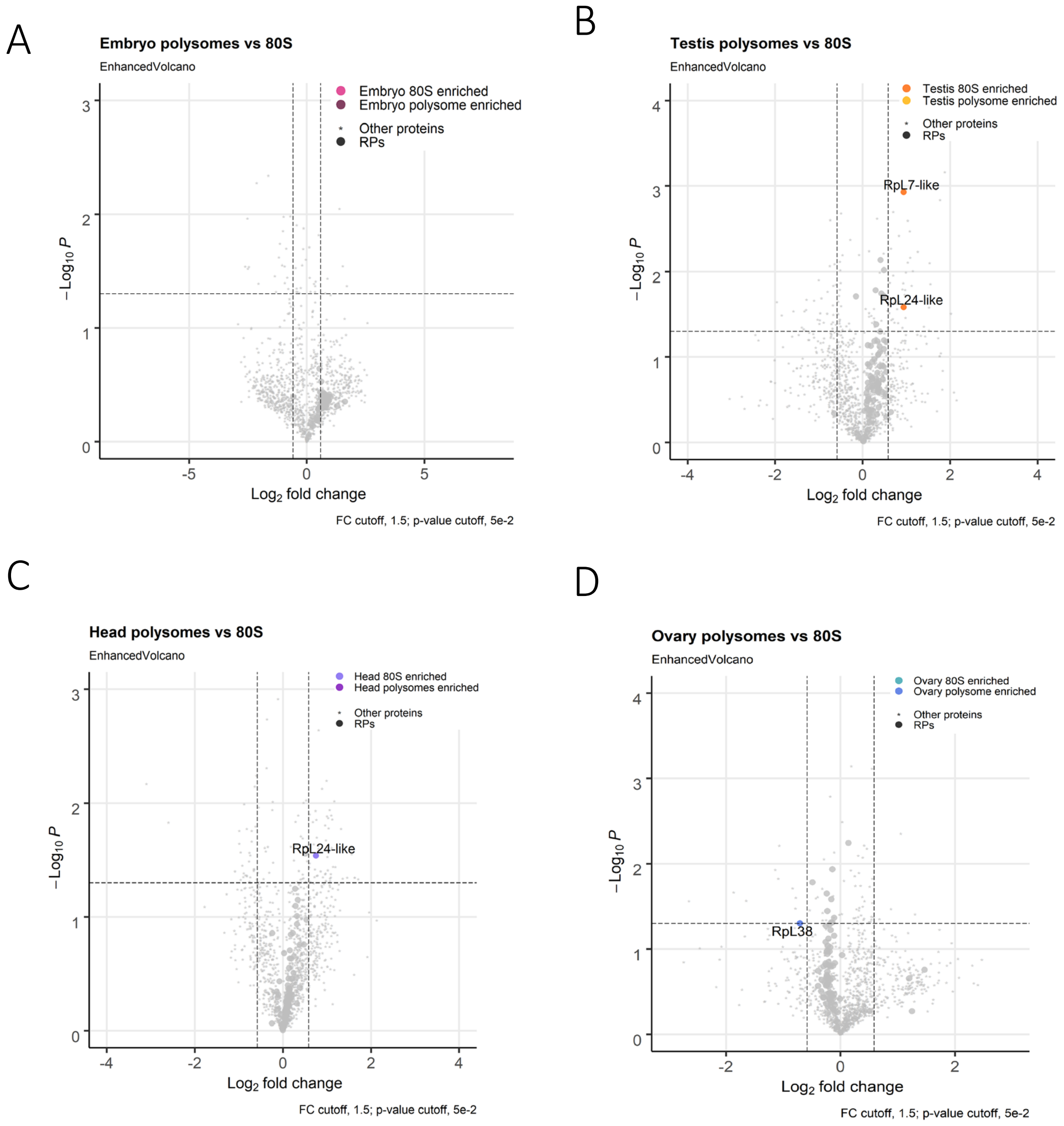

Sup 8: Little difference between composition of 80S and polysome ribosomes
Volcano plots comparing proteins detected in the 80S and polysome fractions for (A) testis, (B) head, (C) ovary and (D) embryo tissue. Log_2 fold-change cut off is 1.5 and p-value <0.05. Enriched RPs are labelled.

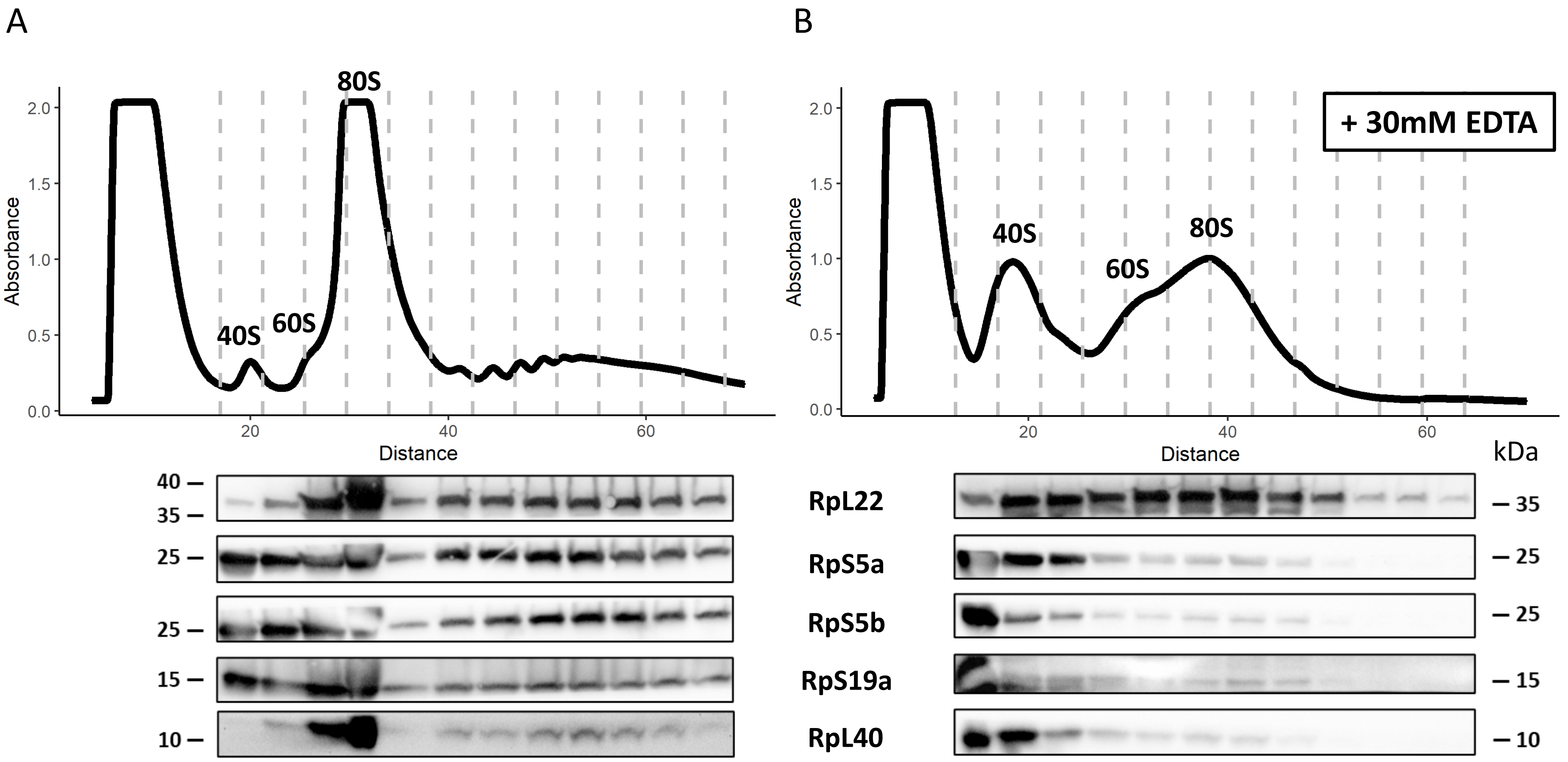

Sup 9: EDTA treatment indicates that enriched paralogs are part of ribosomes
254 nm UV absorbance trace across 18-60% sucrose gradients showing isolated 80S and polysomal complexes from 150 pairs of 3-6 day old adult ovaries (A) without EDTA and (B) with 30 mM EDTA. Fractions were collected (grey lines) and subjected to western blot analysis using paralog specific antibodies. Distance refers to distance (in mm) down sucrose gradient, 0 being top of gradient (18%).

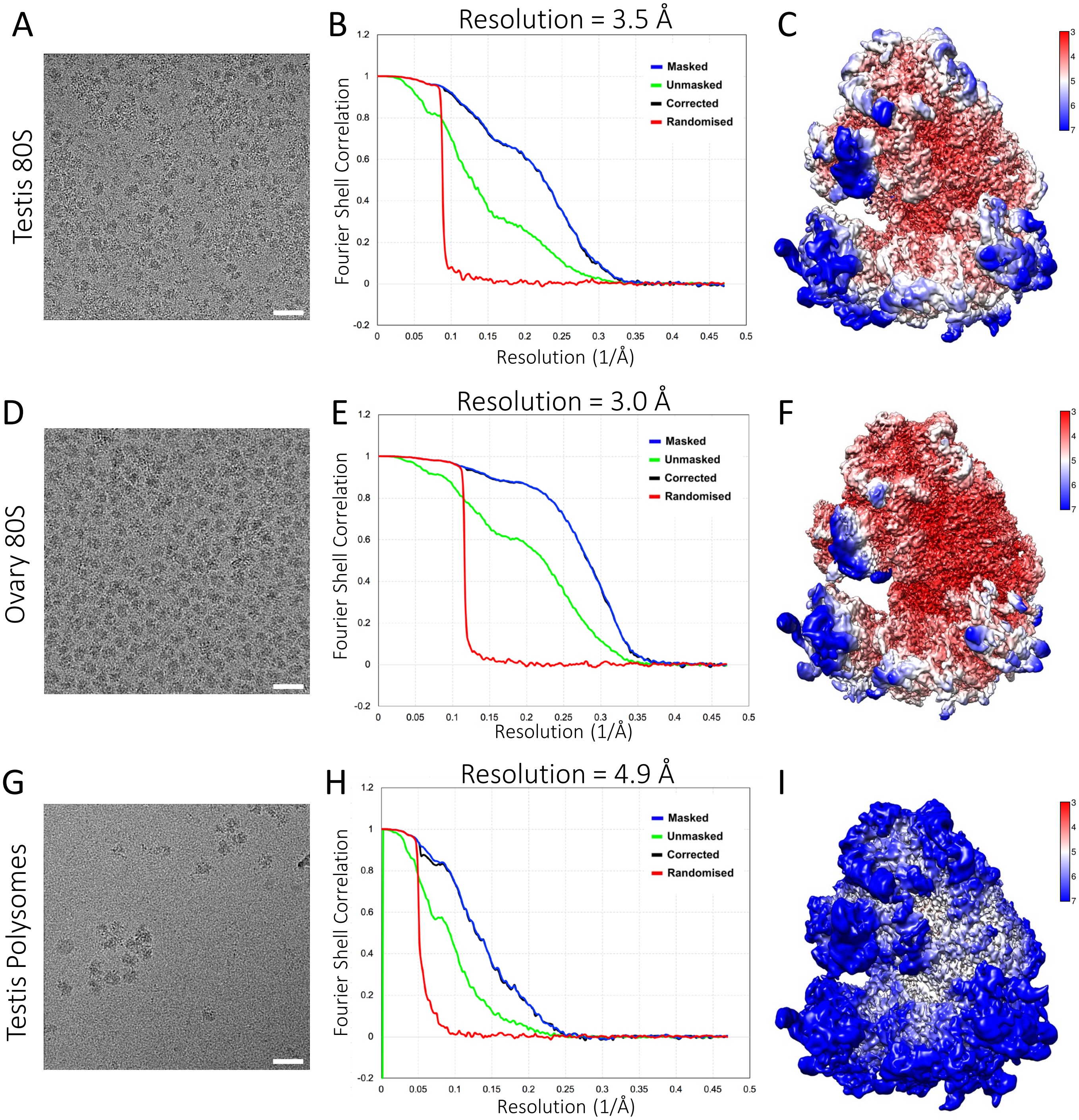

Sup 10: Cryo-electron microscopy of testis and ovary ribosomes

Cryo-electron micrographs (A, D and G), FSC curves (B, E and H) and local resolution coloured maps (C, F and I) for the cryo-EM averages of testis 80S (A-C), ovary 80S (D-F) and testis polysomes (G-I). Scale bars for A, D & G are 50 nm.

A

```
Rabbit_IFRD2      MPRARKGNTPRKGGQRRGGGARSSAQADSGSSEDEAASEARSTTSECPSLLSTTAEDSLG  60
Dmel_IFRD1        MPRRNKKSAA--------GRGRTN----DSNSEDESFDN-VSVYSHMSEVASSE------  41
                  *** .* .:         * .*:.    ...****: .:  *. *.  .: *:

Rabbit_IFRD2      GDTVDEQGPQEDLEEKLKEYVDGLTDKSAKTRQGALESLRLALASRLLPDFLLERRFTLA  120
Dmel_IFRD1        ---ATDELANERFEEKFEKALEQATEKSAQTRVQALQAICELLMHRYMPDFVEDRKMTLM  98
                     . ::  :* :***::: ::  *:***:**  **:::   *  * :***: :*::**

Rabbit_IFRD2      DALEKCLKKGKGEEQALAAAVLGLLCVQLGPGPKGEELFHSLQPLLLSVLSDSTASPAAR  180
Dmel_IFRD1        DFVEKSIRRGKGQEQVWGARLAPLLVLQMGGDE---GISKAMNQFLLNTVQDKSVGFDAR  155
                  * :**.:::***:**. .* :  ** :*:* .     : :::: :**..:.*.:..  **

Rabbit_IFRD2      LHCASALGLGCYVAAADVQDLVSCLACLEGVFSRSCGTGSSTSHVAPAS-LHGVCCAALQ  239
Dmel_IFRD1        AKCCTAVGLLSFLGCEDVGELVHLMQSFEAIFAGSYLRGDDKTPVSVTAEAGTFHAEALN  215
                   :*.:*:** .::.. ** :**  : .:*.:*: *   *...: *: ::    . . **:

Rabbit_IFRD2      AWALLLTICPSAHISHILDRQL------PRLPQLLSSESVNLRIAAGETIALLFELARDL  293
Dmel_IFRD1        AWGLLLTLIPSGDFVSLMTTGQNMFPSIKKFLGLLQSTHLDVRMAAGETIALILESGRAH  275
                  **.****: **..:  ::           ::  **.*  :::*:********::* .*

Rabbit_IFRD2      EEDFVYEDMEALCSTLRTLATDSNKYRAKADRRRQRSTFRAVLHYVEGGECEEETVRFGL  353
Dmel_IFRD1        EEDFLEDDIAELSEAVKQLATDSHKYRAKRDRKAQRATFRDVLRYLEEDISPEISIRFGT  335
                  ****: :*:  *..::: *****:***** **: **:*** **:*:* . . * ::***

Rabbit_IFRD2      EVLYVDSWARHRVYTSFKEALGSGLHHHLQNNELLRDIFGLGPVLVLDAT-ALKACKISR  412
Dmel_IFRD1        ESLTLDSWSIHHQYSAMCTVMGPGMTSQLQENEFIRDIFQLGPRPTNTGINGNAKVKPTK  395
                  * * :***: *: *:::  .:* *:  :**:**::**** ***  .  .  .    * ::

Rabbit_IFRD2      FEKHLYNAAAFKARTKARSRVRDKRADIL-        441
Dmel_IFRD1        LERHLVNAAAFKARSITRGKNRDKRSAVVT        425
                  :*:** ********: :*.: ****: ::
```

B

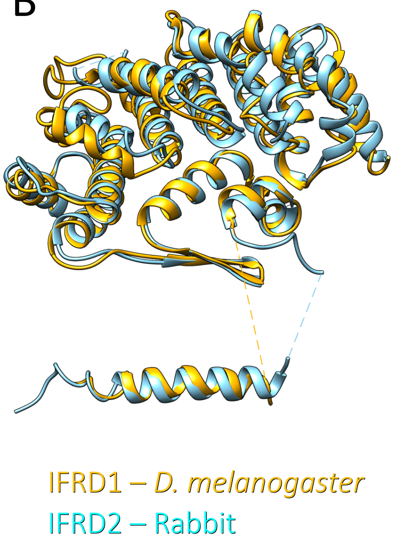

Sup 11: Comparison between rabbit IFRD2 and *D. melanogaster* IFRD1

(A) Clustal-omega alignment of rabbit IFRD2, and *D. melanogaster* IFRD1 protein sequence.

(B) Comparison of the atomic models of rabbit IFRD2 and *D. melanogaster* IFRD1.

Sup 12

A

	Ovary 80S	Testis 80S
**Visual inspection of EM maps and fit of RpL22 and RpL22-like atomic models:**		
RpL22 residues preferred	10	4
RpL22-like residues preferred	2	6
Inconclusive	7	9
**Model-to-map fit after phenix real space refinement:**		
RpL22	0.7588	0.6815
RpL22-like	0.6310	0.7193

B

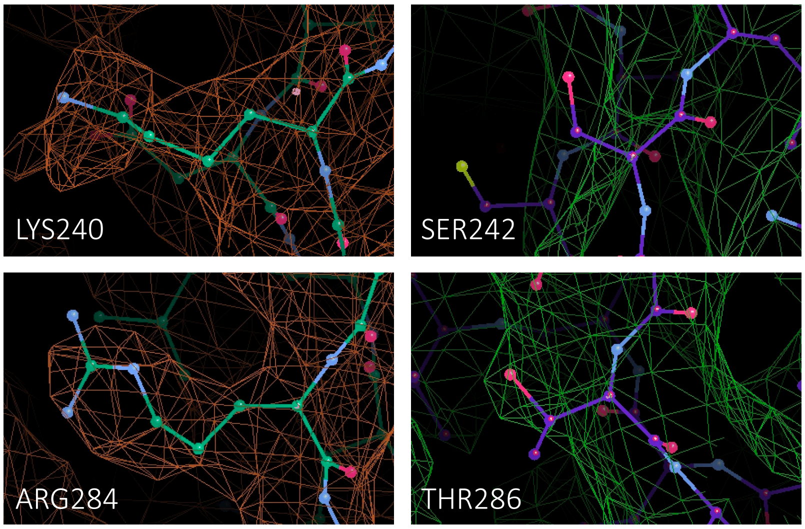

C

Fit of RpL22-like on Testis 80S map:

```
                 240  245  250  255  260  265  270  275  280  285
RpL22_dm         LKLIVSSDVHFSKAYLKYLTKKYLKKNSLRDWIRVVANEKDSYELRYFR
RpL22-like_dm    FSLIIHSGVHFSKRYFKYLTKRYLKKVSLRDWLRVVSTAKDTFAMTYFK
                 :.**: *.***** *:*****:**** *****:***:. **:: : **:
                     245  250  255  260  265  270  275  280  285
```

Fit of RpL22 on Ovary 80S map:

```
                 240  245  250  255  260  265  270  275  280  285
RpL22_dm         LKLIVSSDVHFSKAYLKYLTKKYLKKNSLRDWIRVVANEKDSYELRYFR
RpL22-like_dm    FSLIIHSGVHFSKRYFKYLTKRYLKKVSLRDWLRVVSTAKDTFAMTYFK
                 :.**: *.***** *:*****:**** *****:***:. **:: : **:
                     245  250  255  260  265  270  275  280  285
```

Green: preferred fit; Orange: inconclusive fit

Sup 12: In-depth analysis of density of RpL22/RpL22-like in ovary and testis 80S EM maps
(A) Summary of the fit of residues for RpL22 and RpL22-like into the testis 80S and ovary 80S cryo-EM maps. The model-to-map fit cross-correlation coefficients are also shown (a higher value corresponds with a better fit). (B) Example of the fit of 2 pairs of equivalent residues for RpL22 into the ovary 80S map, and for RpL22-like into the testis 80S map. (C) Schematic result of the fit of RpL22-like into the testis 80S map, and of RpL22 into the ovary 80S map. For each pair of residues that is different between RpL22 and RpL22-like, the one with the best fit is shown in green. If both residues had a similar fit, then the pair is shown in orange (i.e. the fit was inconclusive).

Sup 13

A

	Ovary 80S	Testis 80S
**Visual inspection of EM maps and fit of RpL37a and RpL37b atomic models:**		
RpL37a residues preferred	15	5
RpL37b residues preferred	0	5
Inconclusive	6	11
**Model-to-map fit after phenix real space refinement:**		
RpL37a	0.7379	0.6103
RpL37b	0.6414	0.6217

B

Ovary 80S RpL37a   Testis 80S RpL37b

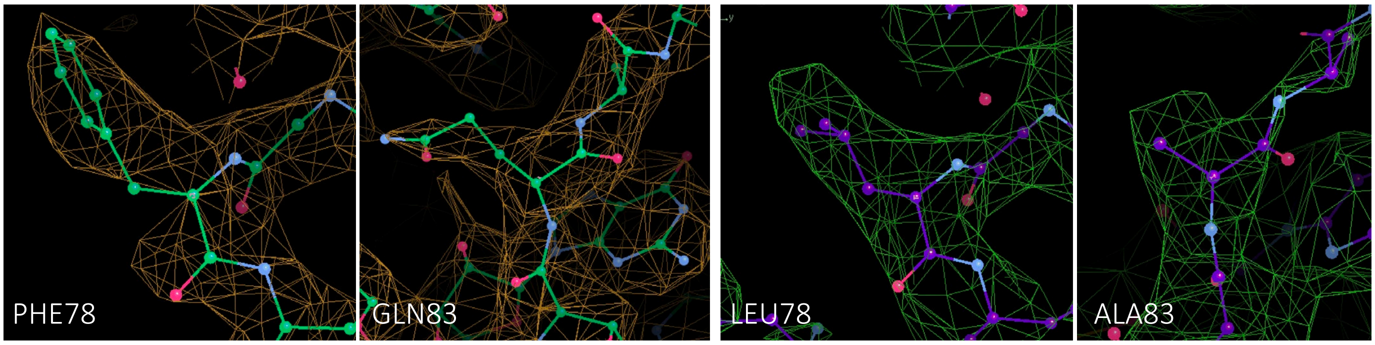

C

Fit of RpL37b on Testis 80S map:

```
                 5    10   15   20   25   30   35   40   45   50   55   60
RpL37a_dm      TKGTSSFGKRHNKTHTLCRRCGRSSYHIQKSTCAQCGYPAAKLRSYNWSVKAKRRKTTG
RpL37b_dm      TKGTTSFGKRHNKTHTICRRCGNSSYHLQKSKCSQCGYPAAKTRSFNWSRKAKGRKAQG
               ****:***********:*****.****:***.*:******** **:*** *** **: *

                 65   70   75   80   85
RpL37a_dm     TGRMQHLKVVRRRFRNGFREGTQAKPKK
RpL37b_dm     TGRMRYLKNLRRRFRNGLREGGAAKKKT
              ****::** :*******:***  ** *.
```

Fit of RpL37a on Ovary 80S map:

```
                 5    10   15   20   25   30   35   40   45   50   55   60
RpL37a_dm      TKGTSSFGKRHNKTHTLCRRCGRSSYHIQKSTCAQCGYPAAKLRSYNWSVKAKRRKTTG
RpL37b_dm      TKGTTSFGKRHNKTHTICRRCGNSSYHLQKSKCSQCGYPAAKTRSFNWSRKAKGRKAQG
               ****:***********:*****.****:***.*:******** **:*** *** **: *

                 65   70   75   80   85
RpL37a_dm     TGRMQHLKVVRRRFRNGFREGTQAKPKK
RpL37b_dm     TGRMRYLKNLRRRFRNGLREGGAAKKKT
              ****::** :*******:***  ** *.
```

Green: preferred fit; Orange: inconclusive fit

Sup 13: In-depth analysis of density of RpL37a/37b in ovary and testis 80S EM maps

(A) Summary of the fit of residues for RpL37a and RpL37b into the testis 80S and ovary 80S cryo-EM maps. The model-to-map fit cross-correlation coefficients are also shown (a higher value corresponds with a better fit). (B) Example of the fit of 2 pairs of equivalent residues for RpL37a into the ovary 80S map, and for RpL37b into the testis 80S map. (C) Schematic result of the fit of RpL37b into the testis 80S map, and of RpL37a into the ovary 80S map. For each pair of residues that is different between RpL37a and RpL37b, the one with the best fit is shown in green. If both residues had a similar fit, then the pair is shown in orange (i.e. the fit was inconclusive).

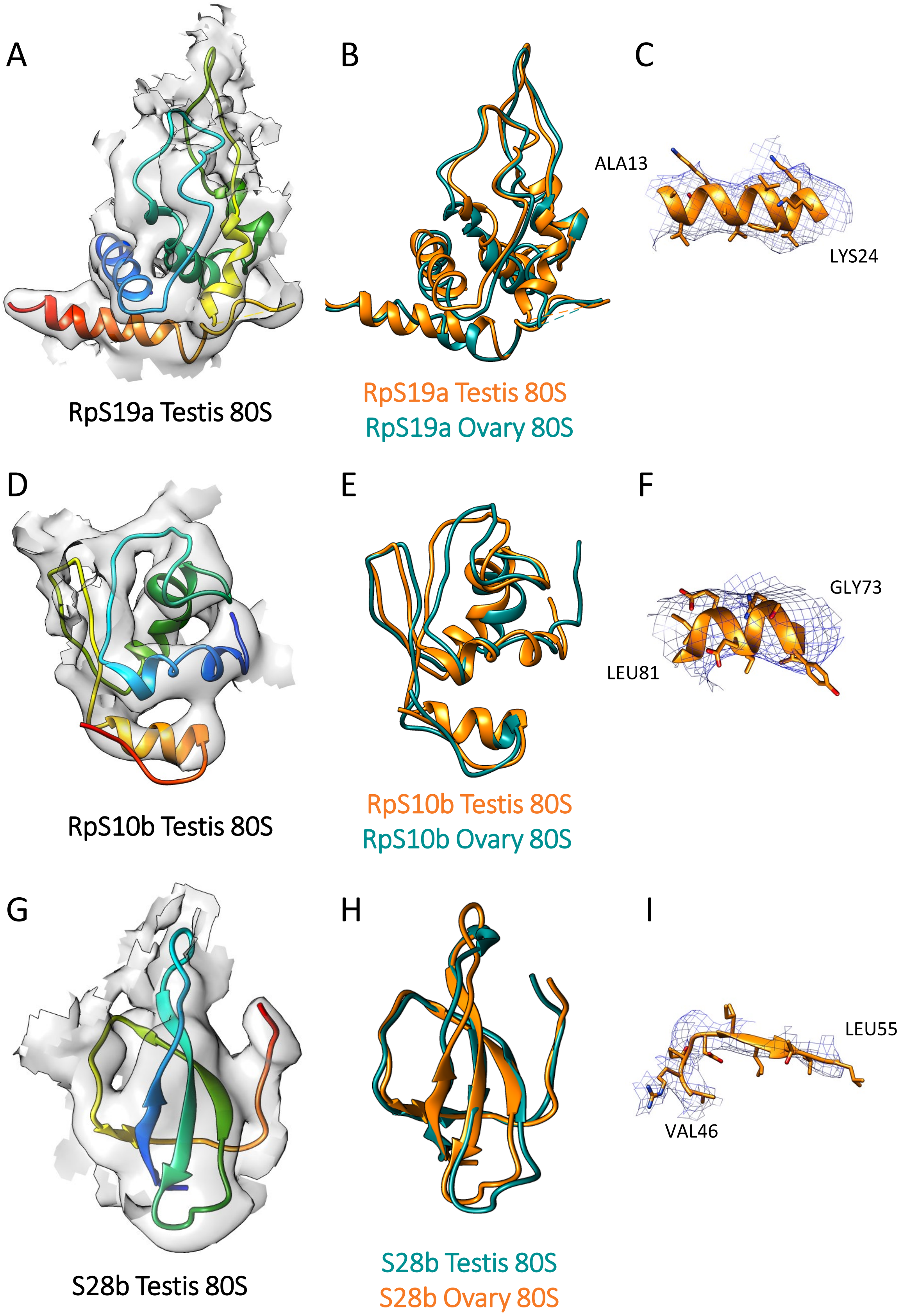

Sup 14: Atomic models of non-switched paralogs with large RMSD between the ovary and testis 80S atomic models

Non-switched paralogs in testis 80S vs ovary 80S are shown. (A-C) RpS19a; (D-F) RpS10b; and (G-I) RpS28b. (A, D and G) Testis atomic models fitted into the EM density. Models are rainbow colored from N-terminus (blue) to C-terminus (red). (B, E and H) Comparison between the testis 80S (orange) and the ovary 80S (teal) atomic models. (C, F and I) Representative fits of the testis 80S atomic models into the EM map.

Sup 15

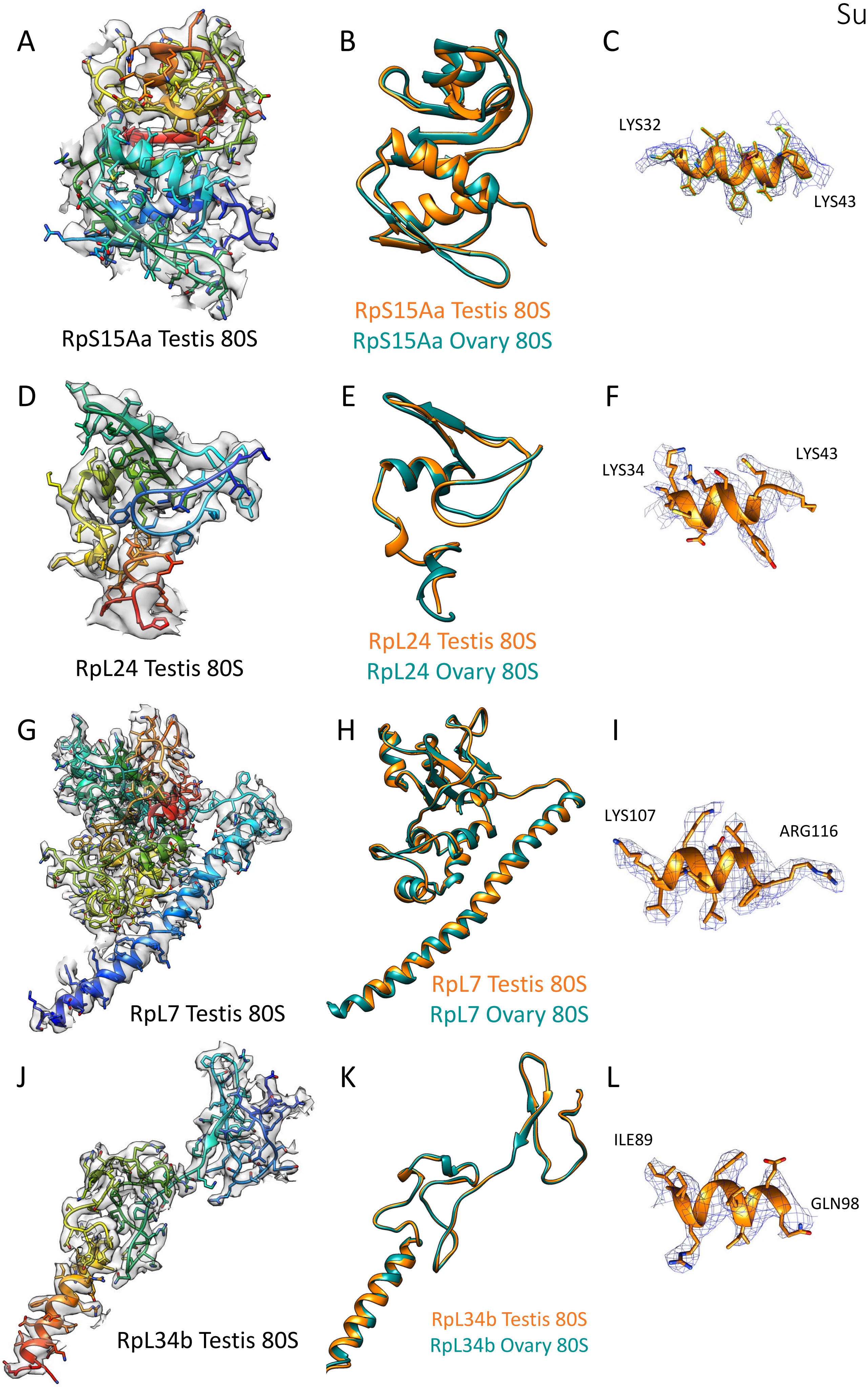
A
RpS15Aa Testis 80S
B
RpS15Aa Testis 80S
RpS15Aa Ovary 80S
C
LYS32
LYS43
D
RpL24 Testis 80S
E
RpL24 Testis 80S
RpL24 Ovary 80S
F
LYS34
LYS43
G
RpL7 Testis 80S
H
RpL7 Testis 80S
RpL7 Ovary 80S
I
LYS107
ARG116
J
RpL34b Testis 80S
K
RpL34b Testis 80S
RpL34b Ovary 80S
L
ILE89
GLN98

Sup 15: Atomic models of non-switched paralogs with low RMSD between the ovary and testis 80S atomic models

Non-switched paralogs in testis 80S vs ovary 80S are shown. (A-C) RpS15Aa; (D-F) RpL24; (G-I) RpL7; and (J-L) RpL34b. (A, D, G and J) Testis atomic models fitted into the EM density. Models are rainbow colored from N-terminus (blue) to C-terminus (red). (B, E, H and K) Comparison between the testis 80S (orange) and the ovary 80S (teal) atomic models. (C, F, I and L) Representative fits of the testis 80S atomic models into the EM map.

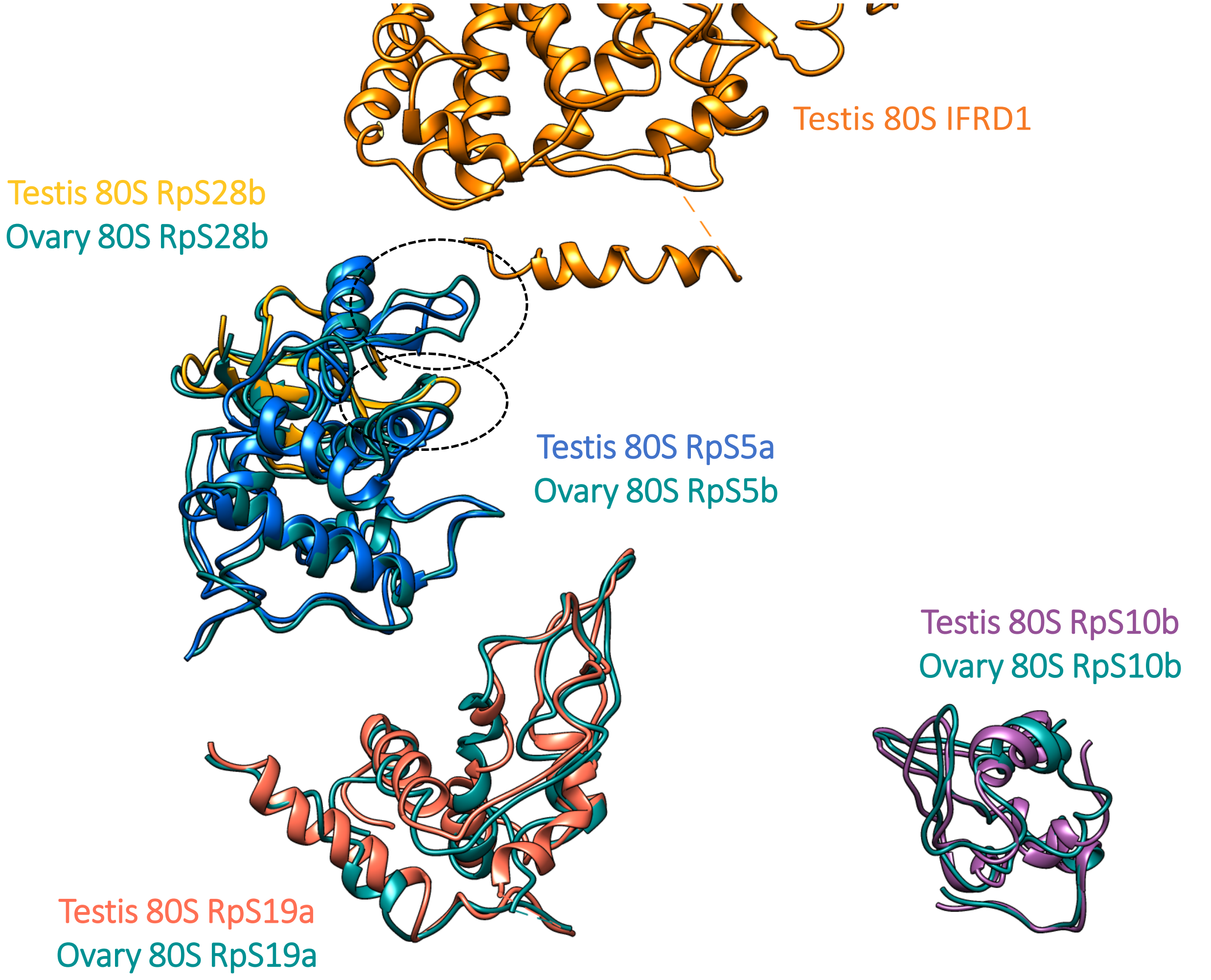

Sup 16: Area around mRNA channel, which in testis 80S is occupied by an alpha-helix from IFRD1
RpS28b (gold), RpS5a (blue) are close to IFRD1 (orange). RpS19a (coral) and RpS10b (purple) are also at the head of the small subunit. Ovary 80S paralogs are superimposed, in teal. The main differences between the PDB models, circled, are in regions close to IFRD1.

Sup 17

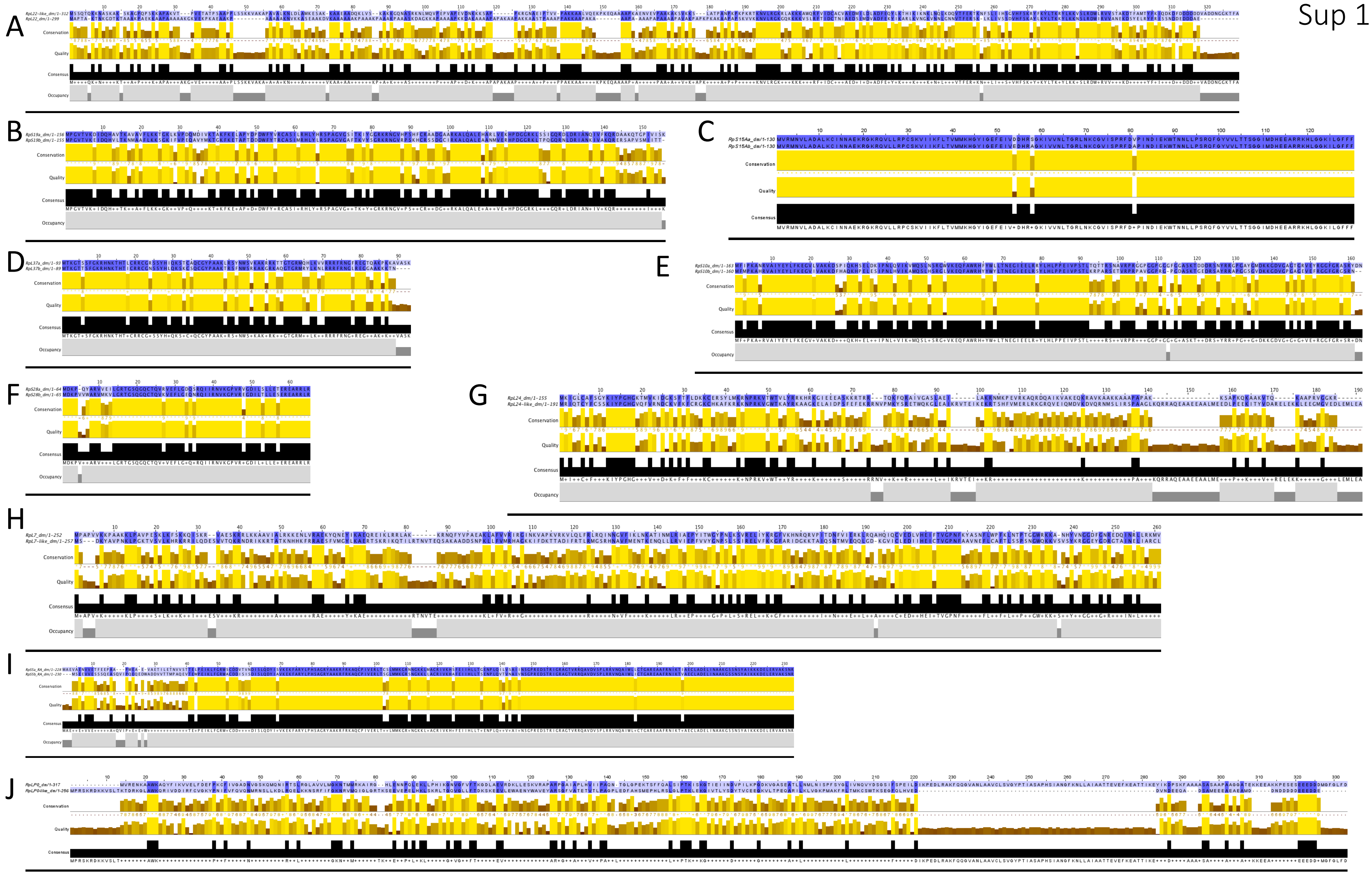

Sup 17: Differences in amino acid sequence between *D. melanogaster* ribosomal paralogs.
Clustal-omega alignment of paralog pairs' protein sequences; A) RpL22/22-like, B) RpS19a/b, C) RpS15Aa/b, D) RpL37a/b, E) RpS10a/b, F) RpS28a/b, G) RpL24/24-like, H) RpL7/7-like, I) RpS5a/b, J) RpLP0/P0-like
