# Supplementary material for: Ribosome heterogeneity in Drosophila melanogaster gonads through paralog-switching
Source: Nucleic Acids Res. 2021 Jul 20;50(4):2240–57. doi: 10.1093/nar/gkab606 (PMC8887423; doi:10.1093/nar/gkab606)
Supplement: gkab606_Supplemental_Files [file gkab606_supplemental_files.zip › Sups10_11.pptx]

## Slide 1
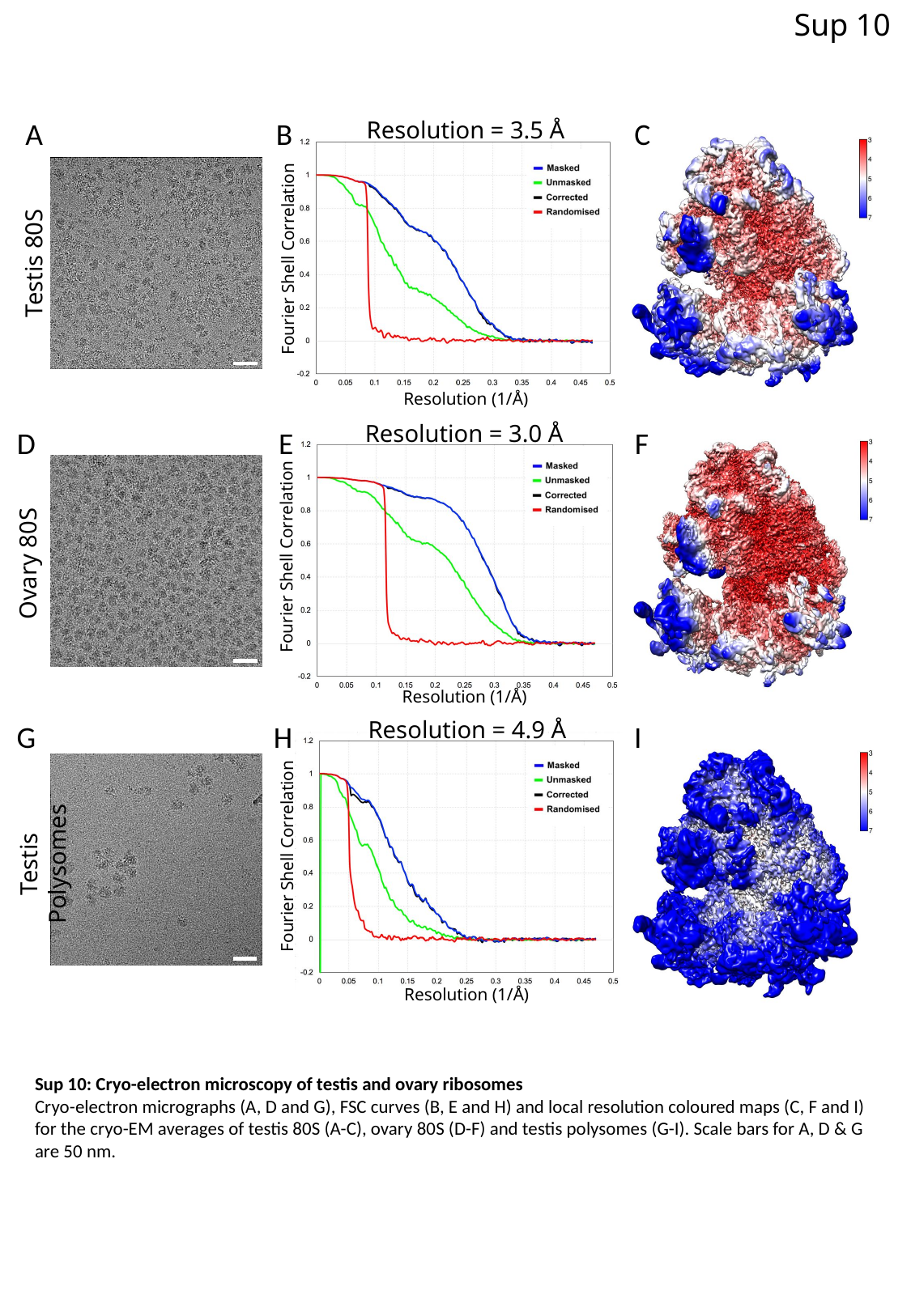

Sup 10
A
B
Resolution = 3.5 Å
C
Fourier Shell Correlation
Testis 80S
Resolution (1/Å)
Resolution = 3.0 Å
D
E
F
Fourier Shell Correlation
Ovary 80S
Resolution (1/Å)
Resolution = 4.9 Å
G
H
I
Fourier Shell Correlation
Testis Polysomes
Resolution (1/Å)
Sup 10: Cryo-electron microscopy of testis and ovary ribosomes
Cryo-electron micrographs (A, D and G), FSC curves (B, E and H) and local resolution coloured maps (C, F and I) for the cryo-EM averages of testis 80S (A-C), ovary 80S (D-F) and testis polysomes (G-I). Scale bars for A, D & G are 50 nm.

## Slide 2
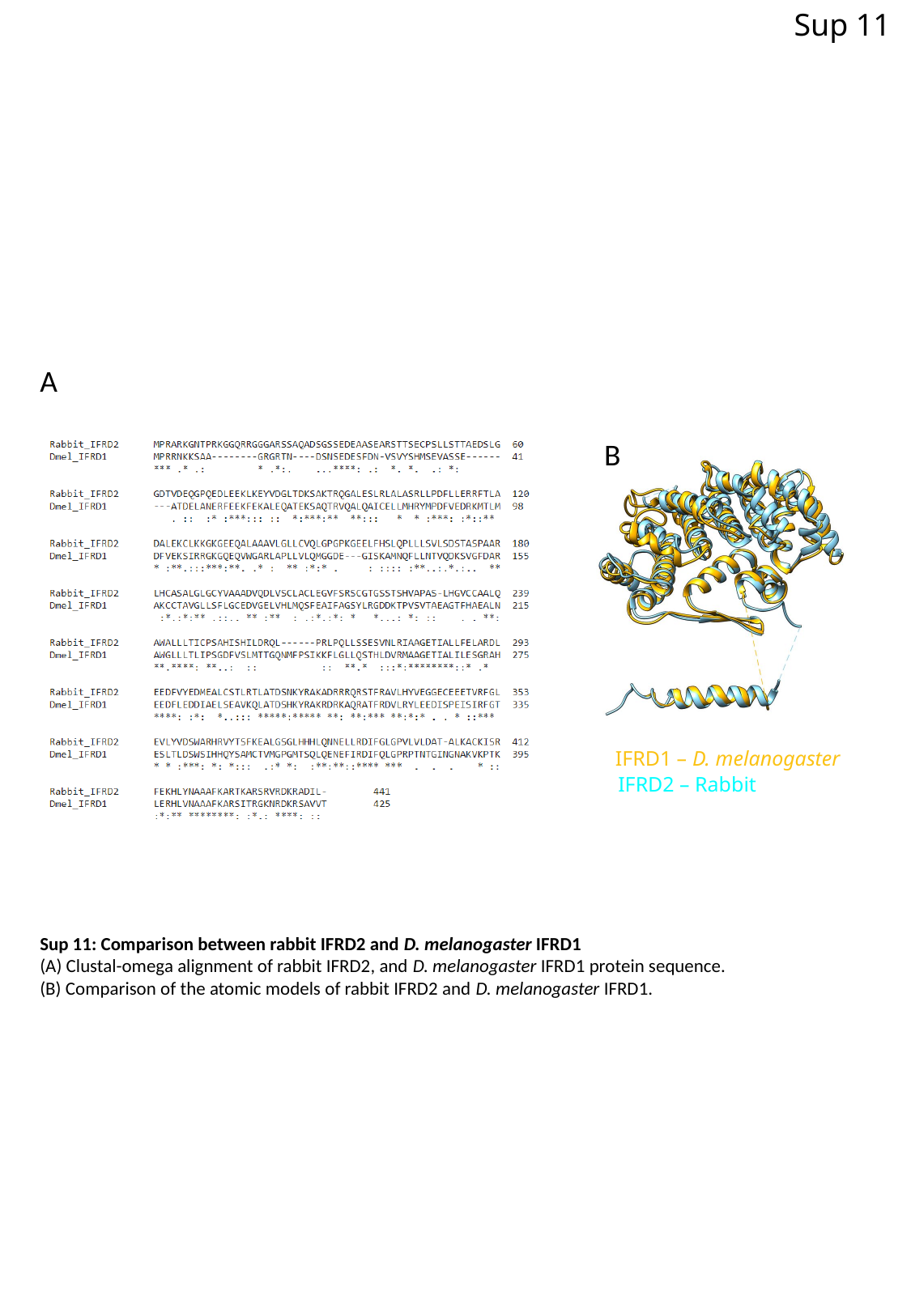

Sup 11
A
B
IFRD1 – D. melanogaster
IFRD2 – Rabbit
Sup 11: Comparison between rabbit IFRD2 and D. melanogaster IFRD1
(A) Clustal-omega alignment of rabbit IFRD2, and D. melanogaster IFRD1 protein sequence. (B) Comparison of the atomic models of rabbit IFRD2 and D. melanogaster IFRD1.
