# Supplementary material for: Ribosome heterogeneity in Drosophila melanogaster gonads through paralog-switching
Source: Nucleic Acids Res. 2021 Jul 20;50(4):2240–57. doi: 10.1093/nar/gkab606 (PMC8887423; doi:10.1093/nar/gkab606)
Supplement: gkab606_Supplemental_Files [file gkab606_supplemental_files.zip › SupTable2.pptx]

## Slide 1
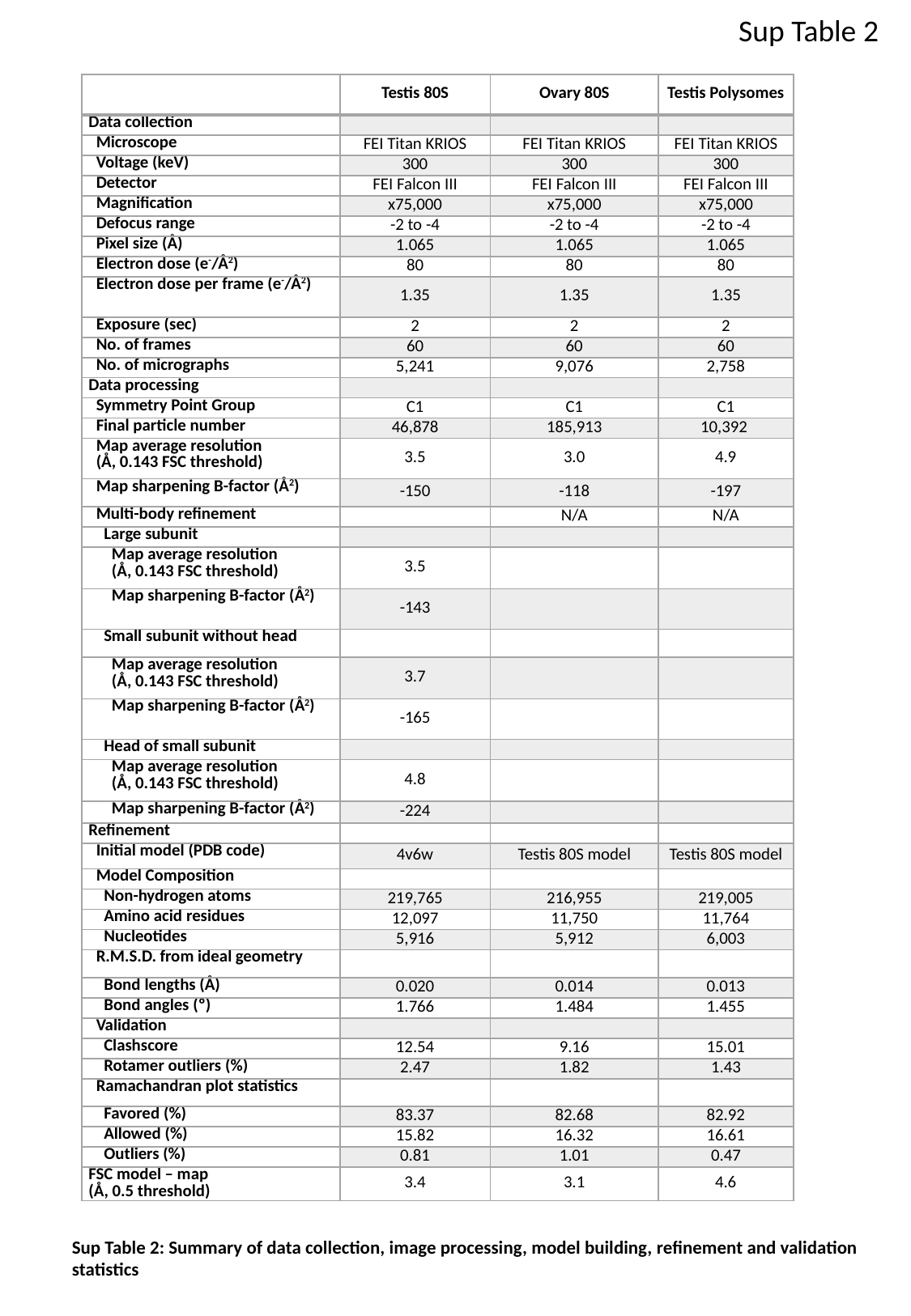

Sup Table 2
| | Testis 80S | Ovary 80S | Testis Polysomes |
| --- | --- | --- | --- |
| Data collection | | | |
| Microscope | FEI Titan KRIOS | FEI Titan KRIOS | FEI Titan KRIOS |
| Voltage (keV) | 300 | 300 | 300 |
| Detector | FEI Falcon III | FEI Falcon III | FEI Falcon III |
| Magnification | x75,000 | x75,000 | x75,000 |
| Defocus range | -2 to -4 | -2 to -4 | -2 to -4 |
| Pixel size (Å) | 1.065 | 1.065 | 1.065 |
| Electron dose (e-/Å2) | 80 | 80 | 80 |
| Electron dose per frame (e-/Å2) | 1.35 | 1.35 | 1.35 |
| Exposure (sec) | 2 | 2 | 2 |
| No. of frames | 60 | 60 | 60 |
| No. of micrographs | 5,241 | 9,076 | 2,758 |
| Data processing | | | |
| Symmetry Point Group | C1 | C1 | C1 |
| Final particle number | 46,878 | 185,913 | 10,392 |
| Map average resolution (Å, 0.143 FSC threshold) | 3.5 | 3.0 | 4.9 |
| Map sharpening B-factor (Å2) | -150 | -118 | -197 |
| Multi-body refinement | | N/A | N/A |
| Large subunit | | | |
| Map average resolution (Å, 0.143 FSC threshold) | 3.5 | | |
| Map sharpening B-factor (Å2) | -143 | | |
| Small subunit without head | | | |
| Map average resolution (Å, 0.143 FSC threshold) | 3.7 | | |
| Map sharpening B-factor (Å2) | -165 | | |
| Head of small subunit | | | |
| Map average resolution (Å, 0.143 FSC threshold) | 4.8 | | |
| Map sharpening B-factor (Å2) | -224 | | |
| Refinement | | | |
| Initial model (PDB code) | 4v6w | Testis 80S model | Testis 80S model |
| Model Composition | | | |
| Non-hydrogen atoms | 219,765 | 216,955 | 219,005 |
| Amino acid residues | 12,097 | 11,750 | 11,764 |
| Nucleotides | 5,916 | 5,912 | 6,003 |
| R.M.S.D. from ideal geometry | | | |
| Bond lengths (Å) | 0.020 | 0.014 | 0.013 |
| Bond angles (º) | 1.766 | 1.484 | 1.455 |
| Validation | | | |
| Clashscore | 12.54 | 9.16 | 15.01 |
| Rotamer outliers (%) | 2.47 | 1.82 | 1.43 |
| Ramachandran plot statistics | | | |
| Favored (%) | 83.37 | 82.68 | 82.92 |
| Allowed (%) | 15.82 | 16.32 | 16.61 |
| Outliers (%) | 0.81 | 1.01 | 0.47 |
| FSC model – map (Å, 0.5 threshold) | 3.4 | 3.1 | 4.6 |
Sup Table 2: Summary of data collection, image processing, model building, refinement and validation statistics
